# Analyzing Big Data in Psychology: A Split/Analyze/Meta-Analyze Approach

**DOI:** 10.3389/fpsyg.2016.00738

**Published:** 2016-05-23

**Authors:** Mike W.-L. Cheung, Suzanne Jak

**Affiliations:** ^1^Department of Psychology, National University of SingaporeSingapore, Singapore; ^2^Department of Methods and Statistics, Utrecht UniversityUtrecht, Netherlands

**Keywords:** big data, multilevel model, structural equation modeling, meta-analysis, R platform

## Abstract

Big data is a field that has traditionally been dominated by disciplines such as computer science and business, where mainly data-driven analyses have been performed. Psychology, a discipline in which a strong emphasis is placed on behavioral theories and empirical research, has the potential to contribute greatly to the big data movement. However, one challenge to psychologists—and probably the most crucial one—is that most researchers may not have the necessary programming and computational skills to analyze big data. In this study we argue that psychologists can also conduct big data research and that, rather than trying to acquire new programming and computational skills, they should focus on their strengths, such as performing psychometric analyses and testing theories using multivariate analyses to explain phenomena. We propose a split/analyze/meta-analyze approach that allows psychologists to easily analyze big data. Two real datasets are used to demonstrate the proposed procedures in R. A new research agenda related to the analysis of big data in psychology is outlined at the end of the study.

The amount of data in the world is enormous. For example, the size of the data that Google owns is estimated to be 15 exabytes [that is 15,000 petabytes or 15 billion gigabytes (GB)], Facebook is estimated to have 150 petabytes of data, and eBay is estimated to have 90 petabytes of data (Huss and Westerberg, 2014). According to IBM, the amount of data produced in the world each day is about 2.5 exabytes. Moreover, the total amount of data in the world is predicted to double every 2 years. Little wonder that big data is a big topic. This is especially the case in the world of business, where data means money. Of course, the value lies not in the data itself, but in the information that can be extracted from the data.

The largest collector of data is probably Google, which uses data from their search engine, for example, to present users with personalized advertisements. Online stores use big data to suggest items that customers might wish to purchase, based on the purchases of customers with similar profiles. Networking sites like LinkedIn and Facebook are excellent at suggesting potential connections for people. With the increasing availability of big datasets, big data has also become a big issue in many scientific disciplines.

Besides the big data available in business and industry, a great deal of large and big data are also freely available to the public (“Open data.” 2015). One of the most important open data initiatives is the open data in government, which makes many government data available over the Internet. For example, the U.S. Government (“Data.gov,” n.d.)^1^ makes more than 195,000 datasets available for downloading. These datasets include topics such as climate, finance, education, and public safety. It is reasonable to expect that more and more big datasets will be freely available in the future. Now the question is whether psychologists know how to analyze these datasets to address important questions in their research domains.

## Examples of large and big data in psychology

Most psychological datasets are relatively small, i.e., small enough to be analyzed using a standard desktop computer. Large datasets occasionally appear in the literature. Examples are the World Values Survey (“WVS Database,” n.d.)^2^, the International Social Survey Programme (ISSP; “ISSP–General information,” n.d.)^3^, the Longitudinal Study of American Youth (LSAY; “LSAY,” n.d.)^4^, the International PISA study (OECD, 2012), and the GLOBE project (House et al., 2004). Taking the World Values Survey as an example, the dataset contains data from 343,309 participants on 1377 variables spanning across 100 regions and six waves. More data are being collected in the coming years. Since many of these datasets are too large, most researchers simply select part of the data in their analyses. As a result, their analyses and interpretations may not be optimally comprehensive.

Big datasets in psychology may also be gathered through online applications, such as “math garden,” which is an online environment in which children can train and develop their mathematical skills (see Klinkenberg et al., 2011). The math garden project collects around 1 million item responses per day, and uses adaptive testing, adjusting the difficulty of the presented items to the estimated ability of the respondent.

Another example of the use of big data in research is an experimental study on visual search by Mitroff et al. (2015). They developed a mobile game in which respondents had to detect illegal items in X-rays of bags, acting as if they were an airport security officer. One of the research goals was to investigate errors in the visual search of (ultra) rare items. The large number of trials available allowed the investigation of visual search regarding very rare events, with targets being presented in 1 out of 1000 trials.

## Characteristics and analysis of big data

### Characteristics of big data

There is no clear consensus on neither who coined the term “Big Data” nor the definition of it (Diebold, 2012). In general one could say big data refers to datasets that cannot be perceived, acquired, managed, and processed by traditional IT and software/hardware tools within a tolerable time (Chen et al., 2014). We adopt this definition on big data. We define large data as datasets that are large in comparison to conventional datasets in psychological research. Researchers can still analyze large datasets with their standard computers but it may take more time to process the data, such that efficient data-analysis is desirable. It should be noted that these definitions are all relative to the computing facilities. A dataset of 10 GB, e.g., the Airlines data in the illustration, is considered as big data in typical computers with 8 GB RAM. The same dataset is no longer big for workstations with 128 GB RAM.

One of the first to describe big data was probably Laney (2001), who used three dimensions, namely *Volume, Velocity*, and *Variety* (the 3 Vs), to describe the challenges with big data. High volume data means that the size of the dataset may lead to problems with storage and analysis. High velocity data refers to data that come in at a high rate and/or have to be processed within as short an amount of time as possible (e.g., real-time processing). High variety data are data consisting of many types, often unstructured, such as mixtures of text, photographs, videos, and numbers.

A fourth V that is often mentioned is *Veracity*, indicating the importance of the quality (or *truthfulness*) of the data (Saha and Srivastava, 2014). Veracity is different in kind from the other three Vs, as veracity is not a characteristic of big data *per se*. That is, data quality is important for all datasets, not only big ones. However, due to the methods that are used to gather big data, the scale of the problems with respect to the veracity of data may be larger with big datasets than with small ones. Therefore, with big data it may be even more important to consider whether the conclusions based on the data are valid than with carefully obtained smaller datasets (Lazer et al., 2014; Puts et al., 2015)

As big data analyses are mainly performed in the physical sciences and business settings, and not commonly in the social sciences, the quality of the data is often not considered in terms of reliability and validity of the constructs of interest, but in terms of screening for duplicate cases and faulty entries. By focusing on the reliability and validity of the data, the veracity of big data is an area where psychology can really contribute to the field of big data. In the illustrations, we demonstrate how reliability and validity can be evaluated in big and large datasets. Example 1 shows how the reliability and the construct validity of the measures can be studied, while Example 2 illustrates how various regression techniques that are often used to study predictive validity, can be applied to big and large datasets.

In order to analyze large volumes of data properly using a typical computer, the size of the dataset cannot be larger than the amount of random-access memory (RAM), which will often be 4 or 8 GB on typical computers. The present study focuses exclusively on how to handle the large volume and the veracity of data in psychology so that psychologists may begin to analyze big data in their research.

## Potential contributions from quantitative psychology and psychology

Psychologists are generally not part of the team in the big data movement (cf. Tonidandel et al., 2015). One of the reasons for their absence may be the high threshold required to take part, e.g., psychologists may have to master new programming skills, and may not have access to big data. In this paper we argue that psychologists are well trained in psychological and behavioral theories, psychometrics, and statistics, that are valuable in understanding big data. They are in a good position to start addressing theory-based research questions with big data.

Psychological theories provide fundamental models to explain behavior. Psychometrics gives us empirical information on the measurement properties of the data (the fourth V, Veracity). Advanced statistics, such as multilevel modeling, structural equation modeling, and meta-analysis, provide statistical methods to test the proposed theories. Psychologists can provide a new perspective on how the data are collected (if it is new), whether the measurements have good psychometric properties, and which statistical models can be used to analyze the data.

Because the systems to manage and query big data require strong computational skills, big data analysis in social sciences calls for interdisciplinary teams of researchers. Specifically, with current big data techniques, psychologists may need to co-work with researchers with knowledge of the big data techniques. However, the technical skills for big data analysis are in high demand and suffer from low supply. It may not be straightforward for a researcher to find a data scientist to work with. Not only because it requires a network to find co-researchers but also because it requires more funding, possibly leading to inequity between well-funded and less well-funded research (Rae and Singleton, 2015). Therefore, although we may all agree that working in an interdisciplinary team that includes substantive researchers with strong theories and data scientists with strong computational skills would be desirable, it may not be possible to form such teams. Thus, it may be wise for psychologists to learn how to analyze their big data.

We outline a simple framework for psychologists to use in analyzing big data. In this framework, psychologists can analyze big data with their favorite statistical models such as regression models, path models, mixed-effects models, or even structural equation models. Therefore, this simple framework provides a stepping-stone for psychologists to analyze big data. Instead of handling all four Vs in big data, this framework focuses solely on the first V (Volume) and the fourth V (Veracity).

The remaining sections are organized as follows. The next section proposes a split/analyze/meta-analyze (SAM) approach to analyze big data. This approach breaks a big data problem into a problem with many smaller and independent pseudo “studies.” Then, meta-analysis is used to summarize the “findings.” We illustrate the proposed method using two empirical datasets. The last section addresses new challenges and future directions related to the proposed approach.

## A SAM approach to analyze big data

In this section we first introduce several statistical platforms to analyze big data and suggest why using R (R Development Core Team, 2016) is a good choice. We review several alternative approaches and explain why these approaches may not be optimal. Common approaches to handling big data are reviewed. We then introduce the proposed SAM approach to analyze big data.

## Statistical platforms for handling big data

There are several statistical platforms and computing languages for analyzing big data. Two popular choices are R and Python (Rossum, 1995). In a survey conducted in the data mining community, R emerged as the second most widely used analytical tool, after a specific data mining tool called “RapidMiner” (Piatetsky, 2014). R comes with many packages to perform statistical analyses that are often applied in psychological research, e.g., multilevel modeling, structural equation modeling, and meta-analysis. R is popular in statistics, while Python is dominant in computer science. The popularity of R is rapidly increasing across many fields (Robert Muenchen, n.d.). It seems legitimate to assume that future psychologists will be more comfortable with R (Culpepper and Aguinis, 2011), especially if they are planning to handle large data. In this paper we will focus on analyses with R, but the general principles apply to Python or other statistical platforms as well.

## Naïve approaches to handling big data

A naïve approach is to handle big data as a typical dataset. This approach, however, rarely works. Because of the large volume of data, most computer facilities cannot hold the data and perform the statistical analyses. A second approach is to analyze only a subset of data. This approach is used by Google for some applications (Bollier, 2010). However, when doing scientific research it is preferable to use as much relevant information as possible. Moreover, conclusions based on a subset of data may be different from those based on the full data, especially when there are geographical clusters or hierarchies in the data.

Another possible approach is to aggregate the data based on some characteristics, e.g., company or geographic locations. Instead of analyzing the raw data, researchers may analyze the aggregated means of the data. This approach was popular in cross-cultural research. For example, the famous cultural dimension of individualism vs. collectivism was derived based on a factor analysis of the means for the country (Hofstede, 1980). The main limitation of this approach is that results based on the raw scores can be totally different from those based on the aggregated scores. Researchers may commit an ecological fallacy by inferring findings from the aggregated scores to the raw scores (Robinson, 1950).

## Common approaches to handling big data

As big data are too big to be directly analyzed, data scientists usually break the data into smaller pieces for parallel analyses. After the analyses, the results are combined (e.g., Chen and Xie, 2012). Two popular programs for parallel computing are MapReduce (Dean and Ghemawat, 2008) and Apache Hadoop (White, 2012). A similar approach is the split-apply-combine approach (Wickham, 2011), which is very popular in R. These approaches involve converting a big dataset into many manageable datasets.

Let us illustrate how the split-apply-combine approach works with a simple example. Suppose we have a dataset on the heights of participants and their countries. We are interested in calculating the mean heights of the participants in each country. The split step groups the heights according to their countries. The apply step calculates the mean height of each country. The combine step merges the mean heights and the countries. Although this example is trivial, more complicated analyses may be used in the apply step. The output of the split-apply-combine approach usually returns a list or a data frame conditioned on the grouping variables. Researchers may apply further calculations on the list of the summaries. In this study, we modify these approaches by using meta-analysis in the last step so that statistical inferences can be made in analyzing big data. The proposed approach is applicable to many statistical analyses.

## The SAM approach

**Figure 1:**
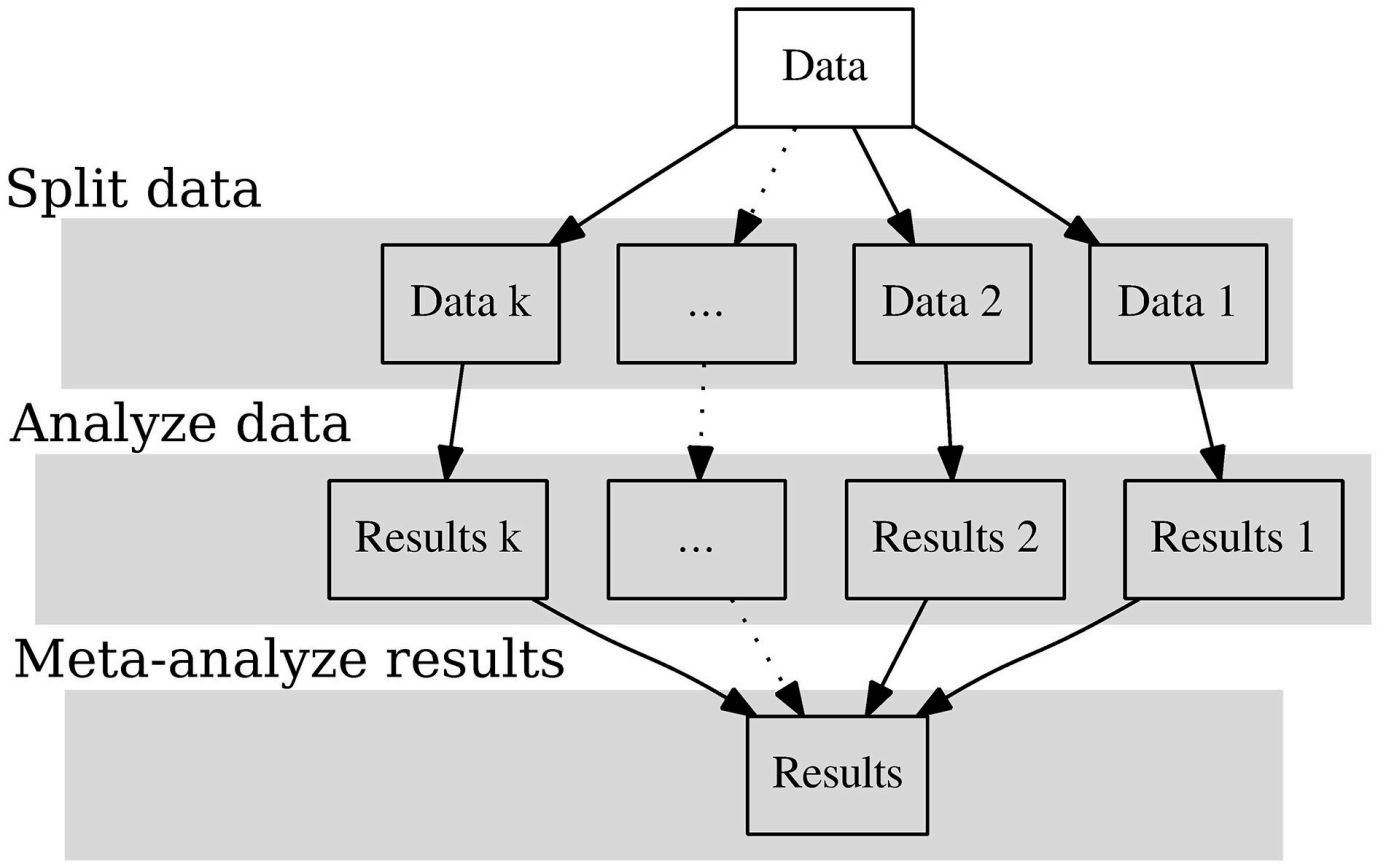
**The split/analyze/meta-analyze (SAM) model**.

Figure 1 shows the graphical representation of the SAM approach. In the first step we split the data into many independent datasets. We treat each dataset as a pseudo “study” and analyze it independently. The parameter estimates are considered as effect sizes in the studies. In the last step the effect sizes are combined with meta-analytic models. The following sections provide more details on the three stages involved.

### Splitting data into many pseudo “studies”

By definition, big data are too big to fit into the computer's RAM. Even if they can be read into the RAM, there may not be sufficient RAM left to perform the analysis. Therefore, we will need to break the dataset into smaller datasets. We propose two methods to split the data depending on the research questions and the data structure. If the data are already stored according to some characteristics, e.g., geographic locations or years, we may split the data based on these characteristics, which is termed *stratified split* here. If there are no special sample characteristics that we can use to split the data, we may apply an arbitrary (random) split on the data, which is termed *random split* here. These two choices have implications for how the results are to be combined in the final step. After splitting the data, each of the resulting datasets can be viewed as a separate pseudo “study.”

### Analyzing data as separate studies

We may apply common statistical analyses, such as regression analysis, reliability analysis, factor analysis, multilevel analysis, or structural equation modeling, on each pseudo “study.” After each analysis, the parameter estimates, e.g., regression coefficients or coefficient alpha, and their sampling covariance matrices are returned. These parameter estimates are treated as effect sizes in the next stage of the analysis. Generally speaking, most parametric techniques, that is, those that result in parameter estimates and a sampling covariance matrix, may be applied in this step. However, two additional points need to be noted. First, it remains unclear how to apply cluster analysis and classifications techniques such as latent class analysis and mixture models. Although we may classify the data into many clusters in each study, future studies may need to address how these clusters are to be combined in the next step.

Second, it is not easy to apply techniques involving model assessment in each pseudo “study.” For example, the illustration using the WVS-dataset presented in the next section shows how to test a one-factor model in the data. The estimated factor loadings are misleading if the proposed model does not fit the data (see Cheung and Cheung, in press for a discussion). In the illustration we address this issue by calculating the correlation matrix as the effect sizes for each study. In the step of combing the results, we apply meta-analytic structural equation modeling (MASEM; Cheung and Chan, 2005; Cheung, 2014) to synthesize the correlation matrices and to test the proposed factor model.

## Combining results with meta-analysis

After obtaining the summary statistics (effect sizes) from various pseudo studies, we may combine them together using meta-analytic models (e.g., Borenstein et al., 2009; Cheung, 2015). It has been found that meta-analysis on summary statistics is equivalent to an analysis of the raw data (Olkin and Sampson, 1998). In fact, random- and mixed-effects meta-analyses are special cases of multilevel models with known sampling variances or covariance matrices (Raudenbush and Bryk, 2002; Hox, 2010; Goldstein, 2011). The proposed approach allows us to study the phenomena at the individual level based on the effect sizes.

If we use a random split in the first stage, the population parameters in different pseudo “studies” are assumed to be equal. All differences in the observed effect sizes are due to sampling error. Therefore, fixed-effects meta-analytic models may be used to combine the parameter estimates. When the “studies” are split according to some characteristics (a stratified split), the population parameters are likely to be different across studies. Besides the differences due to sampling error, there are also true differences (population heterogeneity) across studies. Random-effects models account for the differences between studies, and are more suitable than fixed-effects models in this case (see Hedges and Vevea, 1998 for a discussion of the differences between fixed- and random-effects models).

Suppose that researchers have a big dataset on some purchasing behaviors stratified over products, years, and geographic locations. Researchers are rarely interested in finding one predictive model on the whole data set. Instead, it is more valuable to see how the predictive model works across products, years, and geographic locations. Therefore, the stratified split is usually preferable. In a mixed-effects meta-analysis, the characteristics of the study may be used as predictors to explain variability in the effect sizes. If there is only one effect size, we may use a univariate meta-analysis to summarize the findings. If there are more than one effect sizes, a multivariate meta-analysis or MASEM may be used to summarize the findings (Cheung, 2015).

## Illustrations with two real datasets

We used two datasets to demonstrate how to apply the SAM approach to real data. The first dataset was downloaded from the WVS-website (“WVS Database,” n.d.)^2^. This illustration is useful to show how to analyze large datasets such as ISSP, LSAY, PISA, the GLOBE project, and many Open Data projects. Psychologists may address new research questions based on many large datasets.

The second example was based on airlines data. This dataset was used in the 2009 Data Exposition organized by the American Statistical Association to illustrate how to analyze big data (“2009. Data expo. ASA Statistics Computing and Graphics,” n.d.)^5^. The airlines data are not psychological data, but qualify as big data. Therefore, Example 1 serves to show that we can perform the typical analyses often used in psychological studies with the SAM approach, and allows us to compare (part of) the results with the analysis of the raw data. Example 2 serves to show that the SAM approach also works with truly big data. It also demonstrates the potential contributions of quantitative psychology in analyzing big data. Since the sample sizes are by definition huge in big data, researchers should not solely rely on testing the significance of the parameter estimates. Researchers should focus on the effect sizes and their confidence intervals (CIs; Cumming, 2014). Although we only report the standard errors (*SE*s) in the illustrations, researchers may easily convert them into the CIs. All the analyses are conducted in R. The supplementary documents include annotated R code for all analyses, including the output and figures.

## Example 1: world values survey

The dataset contains the scores of 343,309 participants on 1377 variables spanning 100 regions and 6 waves (1981–1984, 1990–1994, 1995–1998, 1999–2004, 2005–2009, and 2010–2014). One useful tip for handling big data is that it is rarely necessary to analyze all variables. For example, there are 1377 variables in the WVS dataset, but we probably need a handful of them in the analyses. It is crucial not to read irrelevant variables into the RAM so that we can spare more memory for the analyses. We may read subsets of the variables from a database or use some programs to filter the variables before reading the data. The following examples illustrate how to apply the SAM approach.

### Illustration using random split

As we have discussed in this paper, there are two possible methods of splitting large data: via a random split or stratified split based on some sample characteristics. We first illustrate the analysis using a random split.

#### The splitting step

There were a total of 343,309 participants in the data set. To demonstrate the effect of the number of studies on the results, we randomly split the data into *k* = 1, 5, 10, 50, 100, 500, and 1000 studies. When *k* = 1, it simply means that all data were analyzed simultaneously. The choice of *k* is usually arbitrary and depends on the size of the RAM. If *k* is too small, the RAM may not be sufficient for the analysis.

#### The analyzing step

Six variables were selected to illustrate a multiple regression analysis. The dependent variable was life satisfaction (*A*170; 1: *dissatisfied* to 10: *satisfied*), while the predictors were: subjective state of health (*A*009; 1: *very good* to 4: *very poor*), which was reverse coded in the analysis; freedom of choice and control (*A*173; 1: *none at all* to 10: *a great deal*); financial satisfaction (*C*006; 1: *none at all* to 10: *a great deal*); sex (*X*001; 1: *male* and 2: *female*); and age (*X*003). The proposed regression model in the *i*th study is:

(1) $$\begin{array}{l} A170=\beta_{0(\text{i})}+\beta_{1(\text{i})}A009+\beta_{2(\text{i})}A173+\beta_{3(\text{i})}C006+\beta_{4(\text{i})}X001+\\ \;\beta_{5(\text{i})}X003+e \end{array}$$

The subscript *i* indicates that the regression coefficients may vary across studies.

#### The meta-analysis step

After running the regression analysis, the estimated regression coefficients ${\hat{\beta }}_{1\left(i\right)}$ to ${\hat{\beta }}_{5\left(i\right)}$ are available in each study. Since the data were randomly split into *k* studies, the population parameters are assumed to be equal across studies. A multivariate fixed-effects meta-analysis (e.g., Cheung, 2013) is conducted using $y_{1\left(i\right)}={\hat{\beta }}_{1\left(i\right)}$ to $y_{5\left(i\right)}={\hat{\beta }}_{5\left(i\right)}$ as the effect sizes and their sampling covariance matrix *V*_(*i*)_ as the known sampling covariance matrix. We use *y*_(*i*)_ rather than ${\hat{\beta }}_{\left(i\right)}$ to emphasize that the effect sizes are treated as inputs rather than outputs in this step of the analysis.

Table 1 shows the parameter estimates and their *SE*s for the splitting with different numbers of studies. The parameter estimates and their *SE*s are nearly identical. This demonstrates that the SAM approach can recover the relationship at the individual level. Interpretations of the parameter estimates of the SAM approach are identical to those using a conventional analysis at the individual level.

**Table 1:** **Comparisons between analysis of raw data, and analysis based on a fixed-effects meta-analysis with random splits**.

**Numbers of studies**	**Raw data (*k* = 1)**	***k* = 5**	***k* = 10**	***k* = 50**	***k* = 100**	***k* = 500**	***k* = 1000**
**REGRESSION COEFFICIENTS**							
Subjective state of health (A009)	0.4333	0.4333	0.4333	0.4333	0.4334	0.4330	0.4336
Freedom of choice and control (A173)	0.2313	0.2313	0.2313	0.2313	0.2314	0.2315	0.2322
Financial satisfaction (C006)	0.4243	0.4243	0.4243	0.4244	0.4245	0.4257	0.4259
Sex (X001)	0.1708	0.1707	0.1708	0.1708	0.1705	0.1701	0.1698
Age (X003)	0.0580	0.0580	0.0580	0.0580	0.0581	0.0579	0.0575
**STANDARD ERRORS**							
Subjective state of health (A009)	0.0043	0.0043	0.0043	0.0043	0.0043	0.0043	0.0043
Freedom of choice and control (A173)	0.0015	0.0015	0.0015	0.0015	0.0015	0.0015	0.0015
Financial satisfaction (C006)	0.0015	0.0015	0.0015	0.0015	0.0015	0.0014	0.0014
Sex (X001)	0.0070	0.0070	0.0070	0.0070	0.0070	0.0069	0.0069
Age (X003)	0.0023	0.0023	0.0023	0.0023	0.0023	0.0022	0.0022

Dependent variable is life satisfaction (A170).

### Illustration using a stratified split

We split the data according to their regions and waves. After running the analyses, the effect sizes were combined by either a random- or a mixed-effects meta-analysis.

#### The splitting step

Although it is easy to implement a random split based on a fixed-effects model, this may not be realistic in applied settings. Data are usually nested in some hierarchies. For example, participants in WVS were nested within countries and waves. A better approach is to use a stratified split of the data according to countries and waves. Doing this, the number of studies was 239, with 240–6025 respondents per study.

#### The analyzing step

We illustrate multiple regression analysis, mediation analysis, confirmatory factor analysis, and reliability analysis on different variables of the data. For each analysis, we collect the parameter estimates and associated sampling variances and covariances for each of the studies.

#### The meta-analysis step

Since the studies are different in terms of countries and waves, it is reasonable to expect that each study has its own population parameters. Thus, a multivariate random- or mixed-effects meta-analysis is more appropriate than a fixed-effects analysis (e.g., Cheung, 2013). This means that besides the estimated average population effect sizes, we also estimate the variance component of the heterogeneity of the random effects. A study level variable, like “wave” in this example, can be used to explain some variability in effect sizes.

### Results and discussion

The following sections summarize the various statistical analyses using the stratified split.

#### Multiple regression analysis

The regression model in Equation (1) was fitted in each study. Since the estimated regression coefficients were used as effect sizes in the meta-analysis, we used $y_{1\left(i\right)}={\hat{\beta }}_{1\left(i\right)}$ to $y_{5\left(i\right)}={\hat{\beta }}_{5\left(i\right)}$ to represent the effect sizes. The model for the multivariate meta-analysis is:

(2) $$\begin{array}{l} y_{1(i)}=\gamma_{10}+u_{1(i)}+e_{1(i)}\\ \;\vdots \\ y_{5(i)}=\gamma_{50}+u_{5(i)}+e_{5(i)}, \end{array}$$

where $T^{2}=Var\left({\left[u_{1\left(i\right)},\ldots ,u_{5\left(i\right)}\right]}^{T}\right)$ is the variance component of the random effects, and $V_{i}=Var\;\left({\left[e_{1\left(i\right)},\ldots ,e_{5\left(i\right)}\right]}^{T}\right)$ is the known sampling covariance matrix. We may calculate an *I*^2^ (Higgins and Thompson, 2002) to indicate the degree of between-study heterogeneity to the total variance. For example, the $I_{1}^{2}$ for the first effect size *y*_1_ is

(3) $$I_{1}^{2}={\hat{T}}_{11}^{2}/\left({\hat{T}}_{11}^{2}+{\bar{V}}_{11}\right)\;$$

where ${\hat{T}}_{11}^{2}$ and ${\overset{-}{V}}_{11}$ are the estimated heterogeneity and typical known sampling variance for the first effect size, respectively (e.g., Cheung, 2015).

We may test whether *Wave* predicts the effect sizes by using a multivariate mixed-effects meta-analysis using *wave* as a moderator:

(4) $$\begin{array}{l} y_{1(i)}=\gamma_{10}+\gamma_{11}Wave\;+\;u_{1(i)}\;+\;e_{1(i)}\\ \;\vdots \\ y_{5(i)}\;=\;\gamma_{50}\;+\;\gamma_{51}Wave\;+\;u_{5(i)}\;+\;e_{5(i)}, \end{array}$$

An *R*^2^ type index (Raudenbush, 2009) may be used to indicate the percentage of explanation of the heterogeneity variance by the moderator. For example, the $R_{1}^{2}$ for the first effect size *y*_1_ is

(5) $$R_{1}^{2}=1-{\hat{T}}_{11(1)}^{2}/{\hat{T}}_{11(0)}^{2},$$

where ${\hat{T}}_{11\left(1\right)}^{2}$ and ${\hat{T}}_{11\left(0\right)}^{2}$ are the estimated heterogeneity with and without the moderator, respectively (e.g., Cheung, 2015).

To save space, we will only present the results of the mixed-effects meta-analysis by using wave as the moderator here. The estimated effect regression coefficients (${\hat{\gamma }}_{11}$ to ${\hat{\gamma }}_{51}$) and their *SE*s on predicting the regression slopes are: subjective state of health (A009) = 0.0325 (*SE* = 0.0078), freedom of choice and control (A173) = −0.0088 (*SE* = 0.0041), financial satisfaction (C006) = −0.0194 (*SE* = 0.0072), sex (X001) = −0.0012 (*SE* = 0.0072), and age (X003) = −0.0060 (*SE* = 0.0028). All the regression coefficients were statistically significant at α = 0.05 except for the regression coefficient of sex (X001). The estimated *R*^2^ in predicting the heterogeneity variances on the slopes by wave are 0.0881, 0.0215, 0.0312, 0.0000, and 0.0332. The meanings of the estimated intercepts depend on the scaling of the moderator. We do not report them here to save space. Readers may refer to the online Appendix for the full set of results.

#### Mediation analysis

The following example serves to illustrate how the SAM approach can be used to fit models with mediated effects. A mediation model with life satisfaction (A170) as the dependent variable, freedom of choice and control (A173) as the mediator, and subjective state of health (A009) as the predictor, was hypothesized. The mediation model was fitted on each study by country and wave. The estimated indirect effect and the direct effect were considered as two multiple effect sizes for the multivariate meta-analysis (Cheung and Cheung, in press).

**Figure 2:**
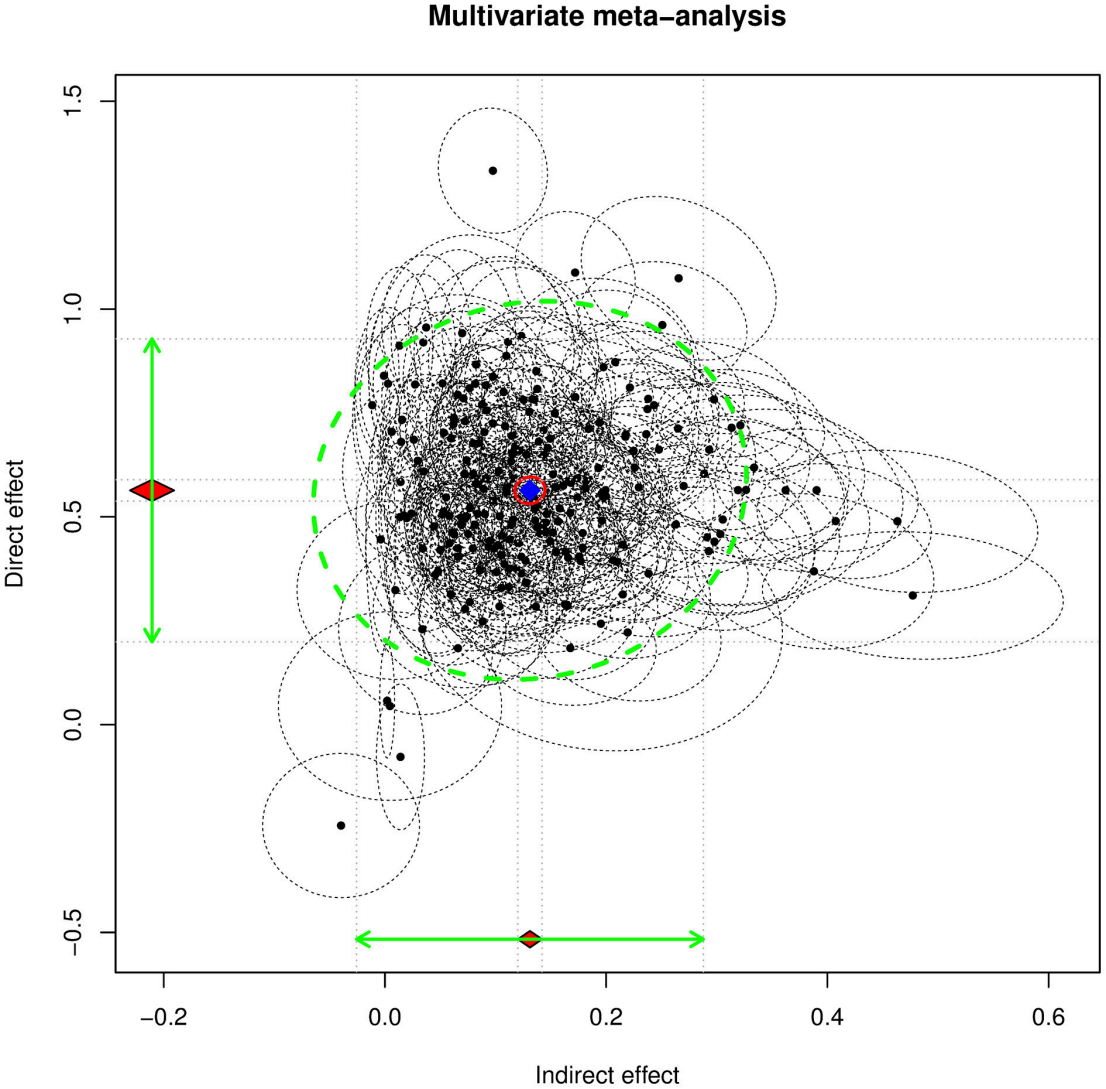
**The 95% confidence ellipse on the indirect and direct effects on the WVS data**.

By running the random-effects meta-analysis, we obtained estimates of the average population indirect and direct effects of 0.1311 (*SE* = 0.0056) and 0.5636 (*SE* = 0.0131), respectively. The estimated heterogeneity variance for the indirect and direct effects is ${\hat{T}}^{2}=\left[\begin{array}{cc} 0.0064\\ 0.0011 & 0.0346 \end{array}\right]$. Figure 2 displays the 95% confidence ellipses (see Cheung, 2013) on the estimated indirect and direct effects. We also conducted a mixed-effect meta-analysis by using wave as a moderator. Wave was significant in predicting the direct effect, 0.0441 (*SE* = 0.0086), *R*^2^ = 0.1206, but not in predicting the indirect effect, −0.0027 (*SE* = 0.0038), *R*^2^ = 0.0058.

#### Confirmatory factor analysis

More advanced multivariate analyses, such as confirmatory factor analysis (CFA), may also be performed with the SAM approach. As an illustration, four items (1: *never justifiable* to 10: *always justifiable*) were used to measure a single factor called “fraud.” These items asked participants whether it was justifiable to (1) claim government benefits to which you are not entitled (F114); (2) avoid paying a fare on public transport (F115); (3) cheat on taxes (F116); and (4) accept a bribe from someone in the course of carrying out one's duties (F117). We used two-stage structural equation modeling (TSSEM; Cheung and Chan, 2005; Cheung, 2014) to fit the one factor model.

Correlation matrices were calculated by country and wave. These correlation matrices were treated as stemming from different studies and averaged together with a random-effects model in the stage 1 analysis. The proposed one-factor model was fitted against the average correlation matrix with its asymptotic covariance matrix as the weight matrix using the weighted least squares estimation method in the stage 2 analysis. The proposed model fits the data reasonable well according to the RMSEA and SRMR with *X*^2^(*df* = 2) = 333.92, *p* < 0.001, RMSEA = 0.0230 and SRMR = 0.0472. The estimated factor loadings with their 95% likelihood-based confidence intervals for the items F114 to F117 were 0.5742 (0.5542, 0.5939), 0.7286 (0.7152, 0.7418), 0.7317 (0.7182, 0.7449), and 0.5852 (0.5659, 0.6041), respectively.

#### Reliability generalization

One of the strong areas in quantitative psychology is to study the measurement properties of measures. In the previous example, we conceptualized four items to measure the concept of “fraud.” We may test the reliability of the scale consisting of these four items by using reliability generalization (e.g., Beretvas and Pastor, 2003; Botella et al., 2010). We first calculated the coefficient alpha and its sampling variance (Bonett, 2010) in each study per country and wave. The estimated coefficient alpha and its sampling variance were tested in a mixed-effects meta-analysis with wave as the moderator. The estimated slope was significant, with 0.0216 (*SE* = 0.005). Wave explains 9.92% of the variation on the coefficient alpha across studies. The estimated residual heterogeneity variance was 0.0087, which is the between-study difference in the reliability coefficient that could not be explained by wave. The estimated coefficient alphas at wave 1 and wave 6 were 0.6342 and 0.7422, respectively.

## Example 2: airlines data

The airlines dataset contains scores on 29 variables from more than 123 million flight records for almost all arrivals and departures at airports in the USA from 1987 to 2008. The sizes of the compressed files and the uncompressed files are 1.7 GB and 12 GB, respectively. Since most big datasets are stored in database format, we simulated this environment by converting the data sets into a SQLite database. This illustrates how the proposed model can be applied to handle other big data. The R code in the online supplement shows how the SQLite database, which is about 14.3 GB in size, was created.

We selected a few variables for this illustration. We were interested in how to predict the *arrival delay time* (in minutes; ArrDelay in the data) by using *departure delay time* (in minutes; DepDelay in the data) and *distance* (Distance in the data) between the original and destination airports (in miles). It was hypothesized that (1) departure delay time was positively related to arrival delay time; while (2) distance between the airports was negatively related to arrival delay time. The logic for the second hypothesis is that a longer travel distance allows the flight to adjust its schedule to compensate for the departure delay.

**Figure 3:**
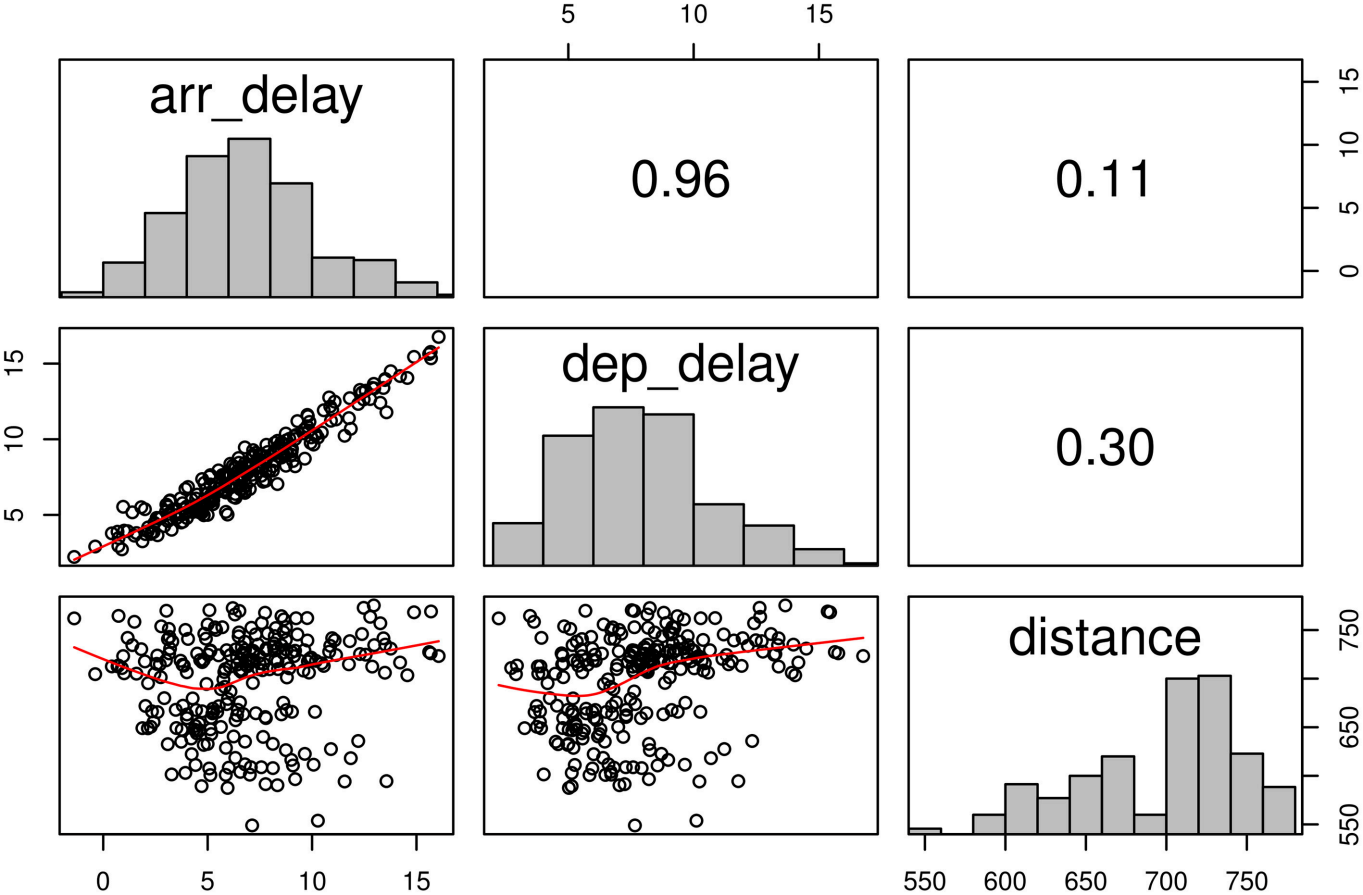
**Scatter plot on the means of the selected variables on the airlines data**.

Before testing the above hypotheses, we reported some descriptive statistics and figures as we usually do in data analyses. As there were too much data, we aggregated the means by years and months. Figure 3 shows the scatter plot on the aggregated *arrival delay time, departure delay time*, and *distance*. As expected, *arrival delay time* and *departure delay time* is positively correlated (*r* = 0.96). However, the direction of the association of *distance* is different from the hypothesized direction (*r* = 0.11). It should be noted that these correlation coefficients were calculated based on the aggregated means. The results may or may not be the same as those based on the individual-level analysis. The SAM approach may correctly analyze the data at the individual level. We applied the SAM approach to test the above hypotheses.

### The splitting step

The data were split by years. There were a total of 22 pseudo “studies,” with sample sizes ranging from 1,311,826 flights in 1987 to 7,453,215 flights in 2007.

### The analyzing and meta-analysis steps

Two different models—a regression model and a mixed-effects model—were considered. Since the scales for arrival delay and distance were very different, distance was divided by 1000 in the analyses.

#### Regression analysis

The regression model assumes that the data within each study are independent. The regression model for the *i*th study was

(6) $$ArrDelay=\beta_{0(i)}+\beta_{Dep(i)}\;DepDelay+\beta_{Dist(i)}\;Distance+e.$$

There is a subscript *i* in the regression coefficients, indicating that they may vary across studies. A multivariate random-effects meta-analysis was used to combine the results. Suppose that the estimated regression coefficients ${\overset{˘}{y}}_{Dep(i)}={\overset{˘}{\beta }}_{Dep(i)}$ and $\;{\overset{˘}{y}}_{Dist(i)}={\overset{˘}{\beta }}_{Dist(i)}$ and its sampling covariance matrix ${\overset{˘}{V}}_{i}$ for the *i*th study are obtained, the multivariate random-effects meta-analysis is:

(7) $$\begin{array}{l} {\overset{˘}{y}}_{Dep(i)}=\gamma_{10}+u_{Dep(i)}+e_{Dep(i)}\\ {\overset{˘}{y}}_{Dist(i)}=\gamma_{20}+u_{Dist(i)}+e_{Dist(i)}, \end{array}$$

where γ_10_ and γ_20_ are the average population effect sizes for ${\overset{˘}{y}}_{Dep(i)}$ and ${\overset{˘}{y}}_{Dist(i)},\;T^{2}=\;Var\left({\left[u_{Dep(i)},u_{Dist(i)}\right]}^{T}\right)$ is the variance component of the random effects, and ${\overset{˘}{V}}_{i}=Var\;\left({\left[e_{Dep(i)},e_{Dist(i)}\right]}^{T}\right)$ is the knownsampling covariance matrix in Equation (6).

We further fitted a multivariate mixed-effects meta-analysis by using year as a moderator:

(8) $$\begin{array}{l} {\overset{˘}{y}}_{Dep(i)}=\gamma_{10}+\gamma_{11}Year+u_{Dep(i)}+e_{Dep(i)}\\ {\overset{˘}{y}}_{Dist(i)}=\gamma_{20}+\gamma_{21}Year+u_{Dist(i)}+e_{Dist(i)}, \end{array}$$

where γ_11_ and γ_21_ are the regression coefficients of predicting the slopes for ${\overset{˘}{y}}_{Dep(i)}$ and ${\overset{˘}{y}}_{Dist(i)}$ by year, which is centered around its mean, and the other quantities are defined similarly to those in Equation (7).

#### Mixed-effects regression analysis

One fundamental assumption underlying the multiple regression model is that the data are independent. This assumption may not be tenable for the data. There may be seasonal effects as the arrival delay is nested within month, day of month, and day of week. Moreover, there may also be locational effects from the airports of origin and destination. With a slight abuse of notation, a mixed-effects model using month, day of month, day of week, origin, and destination as random effects was fitted in each study:

(9) $$\begin{array}{l} ArrDelay\;=\;\beta_{0(i)}\;+\;\beta_{Dep(i)}\;DepDelay\;+\;\beta_{Dist(i)}Distance\\ \;+\;u_{1}\;+\cdots \;+\;u_{5}\;+\;e, \end{array}$$

where *u*_1_···*u*_5_ are the random effects for month, day of month, day of week, origin, and destination, respectively.

After running the mixed-effects analysis, the estimated regression coefficients ${ỹ}_{Dep\left(i\right)}={\tilde{\beta }}_{Dep\left(i\right)}$ and ${ỹ}_{Dist\left(i\right)}={\tilde{\beta }}_{Dist\left(i\right)}$ and their sampling covariance matrix Ṽ_*i*_ are used in a multivariate meta-analysis similar to those in Equations (7) and (8).

### Results and discussion

**Figure 4:**
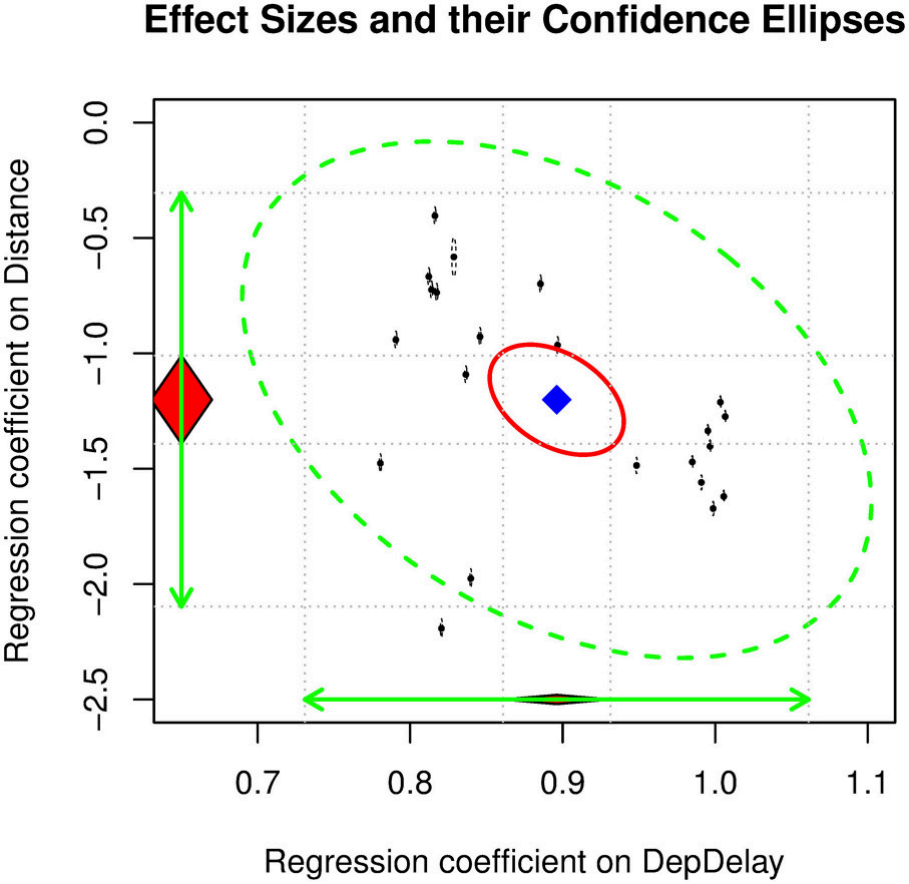
**The 95% confidence ellipse on the regression coefficients on the airlines data**.

Table 2 shows the results of the above analyses. We will only focus on the mixed-effects regression. Comparisons between the results of the regression analysis assuming independence of the observations and the mixed-effects regression analysis will be discussed later. The average effect of departure delay ${\hat{\gamma }}_{10}$ and distance ${\hat{\gamma }}_{20}$ on the arrival delay are 0.8961, and −1.2010, respectively. The *I*^2^ on both effect sizes is almost 1, indicating that there is a large degree of heterogeneity (see Figure 4). These results are consistent with the research hypotheses that departure delay has a positive effect on arrival delay while distance has a negative effect on arrival delay. It should be noted that these results are quite different from those of the analysis of the aggregated means, where the average effects were 1.2084 and −13.1609 for departure delay and distance, respectively.

**Table 2:** **Parameter estimates from the regression model and mixed-effects regression model**.

**Parameter**	**Models used in the analyzing step**			
	**Regression**		**Mixed-effects regression**	
	**Meta-analysis model**		**Meta-analysis model**	
	**Random-effects**	**Mixed-effects (wave as a moderator)**	**Random-effects**	**Mixed-effects (wave as a moderator)**
${\hat{\gamma }}_{10}$	0.9011	0.9011	0.8961	0.8961
*SE*_γ10_	0.0176	0.0105	0.0180	0.0105
${\hat{\gamma }}_{20}$	–0.8624	–0.8623	–1.2010	–1.2009
*SE*_γ20_	0.1048	0.0866	0.0976	0.0773
${\hat{\gamma }}_{11}$		0.0104		0.0108
*SE*_γ11_		0.0017		0.0017
${\hat{\gamma }}_{21}$		–0.0436		–0.0440
*SE*_γ21_		0.0136		0.0122
${\hat{T}}_{11}^{2}$	0.0068	0.0024	0.0071	0.0024
${\hat{T}}_{21}^{2}$	–0.0178	0.0006	–0.0154	0.0037
${\hat{T}}_{22}^{2}$	0.2413	0.1646	0.2091	0.1313
$I_{Dep}^{2}$	1.0000		1.0000	
$I_{Dist}^{2}$	0.9995		0.9991	
$R_{Dep}^{2}$		0.6436		0.6570
$R_{Dist}^{2}$		0.3176		0.3720

${\hat{\gamma }}_{10}$: regression coefficient of Dep. ${\hat{\gamma }}_{20}$: regression coefficient of Dist. ${\hat{\gamma }}_{11}$: regression coefficient of Year in predicting the coefficient of Dep. ${\hat{\gamma }}_{21}$: regression coefficient of Year in predicting the coefficient of Dist.

When year was used as a moderator, the regression coefficients on departure delay ${\hat{\gamma }}_{11}$ and distance ${\hat{\gamma }}_{21}$ were 0.0108 (*SE* = 0.0017), −0.0440 (*SE* = 0.0122), respectively. These findings suggest that the effects of departure delay and distance on arrival delay become stronger over time.

One interesting finding is that the parameter estimates and their *SE*s based on the regression analysis assuming independence of the observations and the mixed-effects regression are comparable. In theory, mixed-effects regression is preferred as it takes the dependence of the data into account. The results seem to suggest that accounting for the dependency is not necessary. A possible reason for this is that the sample sizes are very large in the studies, so the sampling error is very small. Even though the sampling variances are underestimated in the regression analysis by assuming independence of the observations, this bias may not create serious problems in the meta-analysis. However, it should be noted that this is just one example. Further studies are required to clarify whether researchers may “safely” ignore the dependence of data in analyses of big data.

## Issues, new challenges, and research agenda

As we have argued in this paper, the key strength of psychologists is their knowledge of substantive theories, psychometrics, and advanced statistical skills to test research hypotheses. The proposed SAM approach enables psychologists to start analyzing big data. The two illustrations demonstrated how the SAM approach can be used to analyze large behavioral datasets and big data. Existing quantitative methods, such as reliability analysis, multiple regression, mediation analysis, mixed-effects modeling, confirmatory factor analysis, and even structural equation modeling, can be applied to big data. When we move away from laboratory or survey designs to big data, there are many new challenges and issues that need to be addressed. Some of these issues are highlighted in the following section.

## How far can we generalize the research findings in big data analysis?

Sampling error is unlikely to be a major issue in big data analysis because of the large sample size, but sampling bias (selection bias) may be a serious concern. When data are obtained through the Internet or other media, researchers rarely have control over who is providing the data. With data gathered from mobile applications or apps, for example, few or no background characteristics of the respondents may be available to the researchers, making it unclear which population the sample is actually taken from Hargittai (2015). Examples of concerns about Internet samples include the fear that Internet participants are less motivated to engage in the task, that they may be less inclined to cooperate, and that samples obtained through Internet are less diverse than traditional samples.

In a study comparing the characteristics of an Internet sample used for an online questionnaire to those of traditional samples, Gosling et al. (2004) found that Internet samples are not that different from other samples used in psychological research. They concluded that “Internet samples are certainly not representative or even random samples of the general population, but neither are traditional samples in psychology (p. 102).” Due to the size of the sample with big data, it is a concern that even small selection bias may lead to a false rejection of the null hypothesis (Ioannidis, 2005).

Potential data duplication from the same source is another issue. When participants have several email accounts or mobile devices, the data that are collected are not independent. However, researchers may not have the information to link up such data. Ignoring the dependence of the data is a major concern in data analysis. Our airlines data illustration shows that the results of the regression analysis ignoring the dependence and the mixed-effects model taking the dependence of month, day of month, day of week, and airports of the origin and destination into account are comparable. The results seem to suggest that the effect of ignoring the dependence is minor. If this is the case, researchers will be more confident that the potential data duplication may not seriously threaten the quality of big data. Future research should be conducted to verify whether our findings are applicable to other big datasets.

## Which approach, large K (number of studies) or large N (sample size), should we use?

When the data are clustered over some characteristics, e.g., geographic locations or time, it makes sense to split the data according to these characteristics. For example, we analyzed the airlines data by year, leading to 22 groups of data on which the regression models were fitted. We could also analyze the airlines data per year per city of departure, leading to 4461 smaller groups of data. Conducting this analysis lead to estimation problems in the groups with small airports, where the variation in “Distance” was small, e.g., 25% of the airports served no more than 10 destinations. Therefore, when data are split based on specific characteristics, one should be careful not to create groups of data that are too small to obtain reliable results.

When we apply a random split on a dataset, at least two approaches can be used: large *k* with small *N* or small *k* with large *N*. In all cases with groups of equal size, N^*^k is equal to the total number of cases. The main difference is whether we put the computational burden on the primary analysis (the analyzing step) or the meta-analysis. If *N* per study is too large, there may not be sufficient RAM to conduct the analysis. Our findings in Table 1 show that the results are nearly identical with *k* = 1 to *k* = 1000. The choice therefore only depends on the size of the RAM.

Another factor to consider is that meta-analytic techniques are used to combine the parameter estimates. In meta-analysis, reasonably large samples are required in each study so that the parameter estimates are approximately distributed as multivariate normal. Therefore, it is preferable to have at least *N* = 100 per study. However, this requirement will not be a problem with big data.

## How can we address the issue of variety in big data?

We focused on quantitative data in this paper, but different types of data could be informative in psychological research (the V of Variety). One popular application is the analysis of text data from Twitter, which can also be performed through R (Gentry, 2015). These data can for example be used to analyze tweeting behavior of people following shocking events like terrorist attacks (Burnap et al., 2014) and riots (Procter et al., 2013). Another example of big data analysis is the use of Amazon product reviews to evaluate emotional components of attitudes (Rocklage and Fazio, 2015). See for example (Russell, 2013) and (Munzert et al., 2014) for overviews of methods to analyze data from social media sites. In all instances where qualitative data are quantified in the end, and statistical models are to be fitted on the data, the SAM approach as presented in the current article might be useful. These qualitative data provide rich information for psychologists to test theories in real life scenarios rather than in laboratory settings.

## How can we address the issue of velocity in big data?

Another V defining big data is Velocity. In many big data projects, huge amounts of new data are added to the system in real time. It is fair to say that this type of data presents real challenges to psychologists. Researchers often have neither the theories nor the computational techniques to handle this type of data. This issue will become crucial if psychological researchers want to test theories with dynamic data. One possible approach is to regard the new data as if they were new studies in a meta-analysis. Cumulative or updating meta-analysis may be used to combine existing parameter estimates in a meta-analysis along with the new studies (e.g., Lau et al., 1995; Schmidt and Raju, 2007).

On the other hand, these new data may create new opportunities for psychologists to propose and test new dynamic theories related to behavior. The question is how to combine the new data or pseudo “studies” with existing meta-analytic findings under the SAM approach. Future research may address how the SAM approach can be combined with the cumulative meta-analytic techniques.

## Does the current quantitative training meet the future needs in the big data movement?

Aiken et al. (2008) carried out a comprehensive survey on doctoral training in statistics, measurement, and methodology in psychology. They found that PhD students receive more training that supports laboratory research (e.g., ANOVA) than field research (e.g., SEM and multilevel modeling). They were also greatly concerned about the lack of training in measurement in doctoral programs. Generally speaking, quantitative training lags behind the advances in statistical methods. In a slightly different context, Putka and Oswald (2015) discussed how industrial and organizational psychologists should reshape training to meet the new challenges of big data in an organizational environment.

The proposed SAM approach allows researchers to use many of the existing quantitative techniques to analyze big data. It can be fitted easily into the statistical training in psychology. It can serve as a stepping stone for researchers learning how to analyze big data. However, the analysis of big data is still statistically and computationally demanding because researchers are expected to have a knowledge of various advanced statistical techniques and R or some other statistical language suitable for analyzing big data. It is clear that the current quantitative training in psychology is insufficient to meet future demands in the handling of big data analyses, and that to be part of the big data movement, it will be essential to acquire some new skills (Oswald and Putka, 2015). Future studies are definitely required to see how graduate training programs should be reformed to meet these new needs.

## Concluding remarks

Big data opens up many new opportunities in the field of psychology. Researchers may test theories on huge datasets that are based on real human behavior. On the other hand, big data also presents challenges to current and future psychologists. With the SAM approach presented in this study, we aimed to lower the threshold for engaging in big data research.

## Author contributions

MC formulated the research questions and proposed methodology. Both MC and SJ contributed to the data analysis and drafting the paper. Both authors agreed to submit to the Frontiers.

## Funding

MC was supported by the Academic Research Fund Tier 1 (FY2013-FRC5-002) from the Ministry of Education, Singapore. SJ was supported by Rubicon grant 446-14-003 from the Netherlands Organization for Scientific Research (NWO).

### Conflict of interest statement

The authors declare that the research was conducted in the absence of any commercial or financial relationships that could be construed as a potential conflict of interest.

## Footnotes

^1^Retrieved April 12, 2016, from http://www.data.gov/

^2^Retrieved April 13, 2016, from http://www.worldvaluessurvey.org/wvs.jsp

^3^Retrieved May 3, 2015, from http://www.issp.org/

^4^Retrieved May 3, 2015, from http://lsay.org/

^5^Retrieved April 17, 2015, from http://stat-computing.org/dataexpo/2009/
