# Supplementary material for: Analyzing Big Data in Psychology: A Split/Analyze/Meta-Analyze Approach
Source: Front Psychol. 2016 May 23;7:738. doi: 10.3389/fpsyg.2016.00738 (PMC4876837; doi:10.3389/fpsyg.2016.00738)
Supplement: Supplementary file 1 [file DataSheet1.pdf]

Illustrations with the World Values Survey data in R

Mike Cheung and Suzanne Jak

12 May 2016

Contents

Introduction **1**

Data preparation **1**

Installing the R packages 2

Preparing the dataset 2

Multiple regression: Fixed-effects model **2**

Multiple regression and mediation analysis: Random-effects models **7**

Multiple regression 9

Mediation analysis 11

Confirmatory factor analysis and reliability generalization: Random-effects models **16**

Confirmatory factor analysis using the TSSEM approach 17

Reliability generalizability with a random-effects model 22

Settings of the R system **24**

Introduction

- This is an appendix in Cheung and Jak (2016).
- Cheung, M. W.-L., & Jak, S. (2016). Analyzing big data in psychology: A split/analyze/meta-analyze approach. *Frontiers in Psychology*, *7*(738). http://doi.org/10.3389/fpsyg.2016.00738
- The updated version is available at https://github.com/mikewlcheung/code-in-articles/tree/master/Cheung%20and%20Jak%202016/.

Data preparation

- Before running the analyses, we need to install some R packages and download the data. The analyses should run fine in computer systems with at least 8GB RAM.

Installing the R packages

- R can be downloaded at http://www.r-project.org/.
- We only need to install the following packages once.

```
## Installing the R packages from the CRAN
install.packages(c("data.table", "lavaan", "semPlot", "metaSEM"))
```

Preparing the dataset

- The dataset is available at http://www.worldvaluessurvey.org/WVSDocumentationWVL.jsp. Users are required to register before downloading the data.
- In this illustration, we use the dataset in the R format (WVS_Longitudinal_1981-2014_rdata_v_2015_04_18.zip).
- The dataset contains data from 343,309 participants on 1,377 variables spanning across 100 regions and 6 waves (1981-1984, 1990-1994, 1995-1998, 1999-2004, 2005-2009, and 2010-2014).
- The sizes of the data in harddisk and in RAM are 1,389 MB and 1,821 MB, respectively.
- The latest version of the data may be slightly different from that used in this illustration.
- The following R code is used to read and clean up the data. The final data set is named `WVS.Rdata` for ease of manipulations.

```
## Library for efficiently handling large data
library("data.table")

## Unzip the downloaded file
unzip("WVS_Longitudinal_1981-2014_rdata_v_2015_04_18.zip")

## Load the data into R
load("WVS_Longitudinal_1981_2014_R_v2015_04_18.rdata")

## Display the size of the dataset
print(object.size(x=lapply(ls(), get)), units="Mb")

## 1895.3 Mb

## Rename the object for ease of data analyses
WVS <- `WVS_Longitudinal_1981_2014_R_v2015_04_18`

## Remove the old one to clean up memory
rm("WVS_Longitudinal_1981_2014_R_v2015_04_18")

## Convert it into data.table for more efficient data analyses
WVS <- data.table(WVS)

## Save the data so that we do not need to read it from raw data each time
save(WVS, file="WVS.Rdata")
```

Multiple regression: Fixed-effects model

- We randomly split the data into k=100 *studies*.
- We regress *satisfaction with your life* (A170) on *subjective state of health* (A009), *freedom of choice and control* (A173), *satisfaction with financial situation of household* (C006), *sex* (X001), and *age* (X003) in each *study*.

- The following figure displays the regression model.

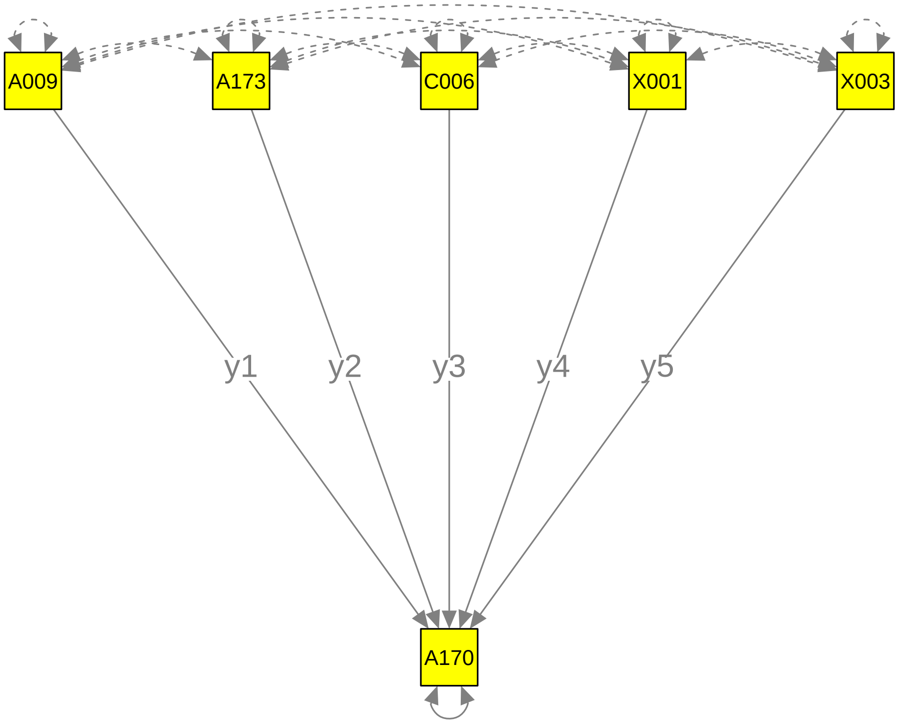

- The estimated regression coefficients with their estimated sampling covariance matrices are treated as multiple effect sizes for a multivariate fixed-effects meta-analysis.
- The variables used in this demonstration are:
  - *State of health (subjective)* (A009): 1 (Very good); 4 (Very poor) (it is reversed before the analyses)
  - *Satisfaction with your life* (A170): 1 (Dissatisfied); 10 (Satisfied)
  - *How much freedom of choice and control* (A173): 1 (None at all); 10 (A great deal)
  - *Satisfaction with financial situation of household* (C006): 1 (None at all); 10 (A great deal)
  - *Sex* (X001): 1 (Male); 2 (Female)
  - *Age* (X003)
  - Negative values in the original dataset represent missing values. They are recoded into missing values (NA) before the analysis.

```
## Load the libraries
library("data.table")
library("lavaan")
library("metaSEM")

## Load the data
load("WVS.Rdata")

## Select the relevant variables to minimize the memory usage
WVS <- WVS[, list(A009, A170, A173, C006, X001, X003, S002, S003)]
```

```
## Reverse coding for A009
## Recode all negative values as NA
## Age (X003) is divided by 10 to improve numerical stability.
WVS[, `:=`(A009 = 5-ifelse(A009 < 0, yes=NA, no=A009),
           A170 =   ifelse(A170 < 0, yes=NA, no=A170),
           A173 =   ifelse(A173 < 0, yes=NA, no=A173),
           C006 =   ifelse(C006 < 0, yes=NA, no=C006),
           X001 =   ifelse(X001 < 0, yes=NA, no=X001),
           X003 =   ifelse(X003 < 0, yes=NA, no=X003/10))]

## No. of studies
k <- 100

## Set seed for replicability
set.seed (871139100)

## Randomly split the data into 100 studies
Study <- sample(1:nrow(WVS)) %% k + 1

## Show the sample sizes in the studies
table(Study)
```

```
## Study
##    1    2    3    4    5    6    7    8    9   10   11   12   13   14   15
## 3412 3413 3413 3413 3413 3413 3413 3413 3413 3413 3413 3413 3413 3413 3413
##   16   17   18   19   20   21   22   23   24   25   26   27   28   29   30
## 3413 3413 3413 3413 3413 3413 3413 3413 3413 3413 3413 3413 3413 3413 3413
##   31   32   33   34   35   36   37   38   39   40   41   42   43   44   45
## 3413 3413 3413 3413 3413 3413 3413 3413 3413 3413 3413 3413 3413 3413 3413
##   46   47   48   49   50   51   52   53   54   55   56   57   58   59   60
## 3413 3413 3413 3413 3413 3413 3413 3413 3413 3413 3413 3413 3413 3413 3413
##   61   62   63   64   65   66   67   68   69   70   71   72   73   74   75
## 3413 3413 3413 3413 3413 3413 3413 3413 3413 3413 3413 3413 3412 3412 3412
##   76   77   78   79   80   81   82   83   84   85   86   87   88   89   90
## 3412 3412 3412 3412 3412 3412 3412 3412 3412 3412 3412 3412 3412 3412 3412
##   91   92   93   94   95   96   97   98   99  100
## 3412 3412 3412 3412 3412 3412 3412 3412 3412 3412
```

```
## Append "Study" into the dataset
WVS[, Study:=Study]

## Set "Study" as the key for grouping
setkeyv(WVS, "Study")

## Function to fit regression analysis
## y1 to y5: Regression coefficients from A170, A009, A173, C006, X001, and X003.
## v11 to v55: Sampling covariance matrix of the parameter estimates
fun.reg <- function(dt) { fit <- try(lm(A170~A009+A173+C006+X001+X003, data=dt), silent=TRUE)

                          ## If there are errors during the analysis, it returns missing values.
                          if (is.element("try-error", class(fit))) {
                            list(y1=NaN,y2=NaN,y3=NaN,y4=NaN,y5=NaN,
                                 v11=NaN,v21=NaN,v31=NaN,v41=NaN,v51=NaN,
```

```
                                       v22=NaN,v32=NaN,v42=NaN,v52=NaN,v33=NaN,
                                       v43=NaN,v53=NaN,v44=NaN,v54=NaN,v55=NaN)
                           } else {
                             ## Extract the regression coefficients excluding the intercept
                             y <- coef(fit)
                             ## Extract the sampling covariance matrix excluding the intercept
                             v <- lav_matrix_vech(vcov(fit)[-1,-1])
                             list(y1=y[2],y2=y[3],y3=y[4],y4=y[5],y5=y[6],
                                  v11=v[1],v21=v[2],v31=v[3],v41=v[4],v51=v[5],
                                  v22=v[6],v32=v[7],v42=v[8],v52=v[9],v33=v[10],
                                  v43=v[11],v53=v[12],v44=v[13],v54=v[14],v55=v[15])
                             }
                           }

########## Split data by "Study" and analyze data with the fun.reg() function on each "Study"
FEM1.reg <- WVS[, fun.reg(.SD), by=list(Study)]

## Show part of the results
head(FEM1.reg)
```

```
##    Study        y1        y2        y3         y4         y5         v11
## 1:     1 0.4172340 0.2472441 0.4166887 0.18381914 0.02613443 0.001943584
## 2:     2 0.4611036 0.2299132 0.4372900 0.07574541 0.05070009 0.001850344
## 3:     3 0.4840781 0.2180822 0.4305115 0.15025652 0.08443550 0.001874817
## 4:     4 0.4367183 0.2135547 0.4151317 0.18799371 0.07226578 0.001929387
## 5:     5 0.4317655 0.2386997 0.4117610 0.16551637 0.06303773 0.001688562
## 6:     6 0.4569928 0.2234663 0.4309035 0.02990574 0.02784601 0.001764056
##              v21           v31          v41          v51          v22
## 1: -8.309871e-05 -0.0001485747 0.0001884341 0.0002996525 0.0002227032
## 2: -7.026020e-05 -0.0001365603 0.0001993570 0.0002644988 0.0002270086
## 3: -7.313360e-05 -0.0001708280 0.0001704147 0.0002951250 0.0002275445
## 4: -8.143360e-05 -0.0001564048 0.0002265432 0.0002856373 0.0002368035
## 5: -8.091491e-05 -0.0001056602 0.0002239312 0.0002577971 0.0002298147
## 6: -7.421872e-05 -0.0001302892 0.0002535206 0.0002962435 0.0002395230
##              v32           v42           v52          v33           v43
## 1: -5.649100e-05  4.381821e-05 -6.614984e-06 0.0002128558  3.510466e-05
## 2: -6.593839e-05  1.663411e-05 -3.576725e-06 0.0002103659  1.967535e-05
## 3: -6.394873e-05  2.954931e-06 -1.262202e-05 0.0002194710 -3.239623e-05
## 4: -6.269739e-05  6.706484e-05 -6.070603e-06 0.0002202558 -4.082936e-05
## 5: -6.218454e-05 -1.571006e-06 -7.463032e-06 0.0002088528 -1.771415e-05
## 6: -6.178713e-05  9.489065e-06 -1.026288e-05 0.0002150476 -2.265777e-05
##              v53         v44           v54          v55
## 1: -2.915789e-05 0.005021478  1.211366e-05 0.0005184840
## 2: -2.325082e-05 0.004841140  3.819308e-05 0.0005119052
## 3: -2.981189e-05 0.004837433  6.794178e-05 0.0005053063
## 4: -3.280232e-05 0.004980850 -8.724074e-06 0.0005186820
## 5: -1.762765e-05 0.004806231  4.151940e-05 0.0005030697
## 6: -2.817320e-05 0.004919920  6.040220e-05 0.0005383494
```

```
########## Meta-analyze results with a multivariate fixed-effects meta-analysis:
########## Variance component is fixed at 0: RE.constraints=matrix(0, ncol=5, nrow=5)
FEM2.reg <- meta(y=cbind(y1,y2,y3,y4,y5),
                 v=cbind(v11,v21,v31,v41,v51,v22,v32,v42,v52,v33,v43,v53,v44,v54,v55),
```

```r
                 data=FEM1.reg, RE.constraints=matrix(0, ncol=5, nrow=5),
                 model.name="Regression analysis FEM")
summary(FEM2.reg)
```

```
##
## Call:
## meta(y = cbind(y1, y2, y3, y4, y5), v = cbind(v11, v21, v31,
##     v41, v51, v22, v32, v42, v52, v33, v43, v53, v44, v54, v55),
##     data = FEM1.reg, RE.constraints = matrix(0, ncol = 5, nrow = 5),
##     model.name = "Regression analysis FEM")
##
## 95% confidence intervals: z statistic approximation
## Coefficients:
##             Estimate Std.Error    lbound    ubound z value  Pr(>|z|)
## Intercept1 0.4332823 0.0042807 0.4248923 0.4416723 101.218 < 2.2e-16 ***
## Intercept2 0.2314661 0.0015236 0.2284800 0.2344522 151.925 < 2.2e-16 ***
## Intercept3 0.4243198 0.0014509 0.4214761 0.4271634 292.459 < 2.2e-16 ***
## Intercept4 0.1703349 0.0069530 0.1567073 0.1839625  24.498 < 2.2e-16 ***
## Intercept5 0.0580356 0.0022538 0.0536183 0.0624529  25.750 < 2.2e-16 ***
## ---
## Signif. codes:  0 '***' 0.001 '**' 0.01 '*' 0.05 '.' 0.1 ' ' 1
##
## Q statistic on the homogeneity of effect sizes: 566.5829
## Degrees of freedom of the Q statistic: 495
## P value of the Q statistic: 0.01409095
##
## Heterogeneity indices (based on the estimated Tau2):
##                               Estimate
## Intercept1: I2 (Q statistic)        0
## Intercept2: I2 (Q statistic)        0
## Intercept3: I2 (Q statistic)        0
## Intercept4: I2 (Q statistic)        0
## Intercept5: I2 (Q statistic)        0
##
## Number of studies (or clusters): 100
## Number of observed statistics: 500
## Number of estimated parameters: 5
## Degrees of freedom: 495
## -2 log likelihood: -2145.318
## OpenMx status1: 0 ("0" or "1": The optimization is considered fine.
## Other values may indicate problems.)
```

- As a comparison we also test the regression analysis on all data (N=343,309).

```r
summary( lm(A170~A009+A173+C006+X001+X003, data=WVS) )
```

```
##
## Call:
## lm(formula = A170 ~ A009 + A173 + C006 + X001 + X003, data = WVS)
##
## Residuals:
##     Min      1Q  Median      3Q     Max
```

```
## -8.9722 -1.1023  0.0737  1.1220  8.1854
##
## Coefficients:
##             Estimate Std. Error t value Pr(>|t|)
## (Intercept) 0.860691   0.021981   39.16   <2e-16 ***
## A009        0.433275   0.004282  101.18   <2e-16 ***
## A173        0.231292   0.001524  151.75   <2e-16 ***
## C006        0.424283   0.001451  292.34   <2e-16 ***
## X001        0.170776   0.006956   24.55   <2e-16 ***
## X003        0.057962   0.002255   25.71   <2e-16 ***
## ---
## Signif. codes:  0 '***' 0.001 '**' 0.01 '*' 0.05 '.' 0.1 ' ' 1
##
## Residual standard error: 1.905 on 301818 degrees of freedom
##   (39447 observations deleted due to missingness)
## Multiple R-squared:   0.39,  Adjusted R-squared:  0.3899
## F-statistic: 3.859e+04 on 5 and 301818 DF,  p-value: < 2.2e-16
```

Multiple regression and mediation analysis: Random-effects models

- The data are grouped according to `Wave` and `Country`.
- Random-effects models are used to account for the differences in `Wave` and `Country` and mixed-effects models are also fitted by using `Wave` as a moderator.

```r
## Clear all objects in the work space
rm(list=ls())

## Load the data
load("WVS.Rdata")

## Sample sizes of S002 (Wave) and S003 (Country)
## Please refer to http://www.worldvaluessurvey.org/WVSDocumentationWVL.jsp
## for the country names.
table(WVS[, c("S002","S003"), with=FALSE])
```

```
##     S003
## S002    8   12   20   31   32   36   48   50   51   70   76  100  112  124
##    1    0    0    0    0 1005 1228    0    0    0    0    0    0    0    0
##    2    0    0    0    0 1002    0    0    0    0    0 1782    0 1015    0
##    3  999    0    0 2002 1079 2048    0 1525 2000    0    0 1072 2092    0
##    4 1000 1282    0    0 1280    0    0 1500    0 1200    0    0    0 1931
##    5    0    0 1003    0 1002 1421    0    0    0    0 1500 1001    0 2164
##    6    0 1200    0 1002 1030 1477 1200    0 1100    0 1486    0 1535    0
##     S003
## S002  152  156  158  170  191  196  203  214  218  222  231  233  246  250
##    1    0    0    0    0    0    0    0    0    0    0    0    0 1003    0
##    2 1500 1000    0    0    0    0  924    0    0    0    0    0    0    0
##    3 1000 1500  780 6025 1196    0 1147  417    0 1254    0 1021  987    0
##    4 1200 1000    0    0    0    0    0    0    0    0    0    0    0    0
##    5 1000 1991 1227 3025    0 1050    0    0    0    0 1500    0 1014 1001
##    6 1000 2300 1238 1512    0 1000    0    0 1202    0    0 1533    0    0
##     S003
```

```
## S002  268  275  276  288  320  344  348  356  360  364  368  376  380  392
##    1    0    0    0    0    0    0 1464    0    0    0    0    0    0 1204
##    2    0    0    0    0    0    0    0 2500    0    0    0    0    0 1011
##    3 2008    0 2026    0    0    0  650 2040    0    0    0    0    0 1054
##    4    0    0    0    0    0    0    0 2002 1000 2532 2325 1199    0 1362
##    5 1500    0 2064 1534 1000 1252 1007 2001 2015 2667 2701    0 1012 1096
##    6 1202 1000 2046 1552    0 1000    0 1581    0    0 1200    0    0 2443
##     S003
## S002  398  400  410  414  417  422  428  434  440  458  466  484  498  499
##    1    0    0  970    0    0    0    0    0    0    0    0 1837    0    0
##    2    0    0 1251    0    0    0    0    0    0    0    0 1531    0    0
##    3    0    0 1249    0    0    0 1200    0 1009    0    0 2364  984  240
##    4    0 1223 1200    0 1043    0    0    0    0    0    0 1535 1008 1060
##    5    0 1200 1200    0    0    0    0    0    0 1201 1534 1560 1046    0
##    6 1500 1200 1200 1303 1500 1200    0 2131    0 1300    0 2000    0    0
##     S003
## S002  504  528  554  566  578  586  604  608  616  630  634  642  643  646
##    1    0    0    0    0    0    0    0    0    0    0    0    0    0    0
##    2    0    0    0 1001    0    0    0    0  938    0    0    0 1961    0
##    3    0    0 1201 1996 1127  733 1211 1200 1153 1164    0 1239 2040    0
##    4 1251    0    0 2022    0 2000 1501 1200    0  720    0    0    0    0
##    5 1200 1050  954    0 1025    0 1500    0 1000    0    0 1776 2033 1507
##    6 1200 1902  841 1759    0 1200 1210 1200  966    0 1060 1503 2500 1527
##     S003
## S002  682  688  702  703  704  705  710  716  724  752  756  764  780  788
##    1    0    0    0    0    0    0 1596    0    0    0    0    0    0    0
##    2    0    0    0  466    0    0 2736    0 1510    0 1400    0    0    0
##    3    0 1280    0 1095    0 1007 2935    0 1211 1009 1212    0    0    0
##    4 1502 1200 1512    0 1000    0 3000 1002 1209    0    0    0    0    0
##    5    0    0    0    0 1495 1037 2988    0 1200 1003 1241 1534 1002    0
##    6    0    0 1972    0    0 1069 3531 1500 1189 1206    0 1200  999 1205
##     S003
## S002  792  800  804  807  818  826  834  840  854  858  860  862  887  891
##    1    0    0    0    0    0    0    0    0    0    0    0    0    0    0
##    2 1030    0    0    0    0    0    0    0    0    0    0    0    0    0
##    3 1907    0 2811  995    0 1093    0 1542    0 1000    0 1200    0    0
##    4 3401 1002    0 1055 3000    0 1171 1200    0    0    0 1200    0    0
##    5 1346    0 1000    0 3051 1041    0 1249 1534 1000    0    0    0 1220
##    6 1605    0 1500    0 1523    0    0 2232    0 1000 1500    0 1000    0
##     S003
## S002  894  914
##    1    0    0
##    2    0    0
##    3    0  800
##    4    0    0
##    5 1500    0
##    6    0    0
```

```
## Select the relevant variables to minimize memory usage
WVS <- WVS[, list(A009, A170, A173, C006, X001, X003, S002, S003)]

## Set Wave and Country as key variables for fast reference
## S002: Wave (1 to 6)
## S003: Country
```

```
setkeyv(WVS, c("S002", "S003"))

## Reverse coding for A009
## Recode all negative values as NA
## Age (X003) is divided by 10 to improve numerical stability.
WVS[, `:=`(A009 = 5-ifelse(A009 < 0, yes=NA, no=A009),
           A170 =   ifelse(A170 < 0, yes=NA, no=A170),
           A173 =   ifelse(A173 < 0, yes=NA, no=A173),
           C006 =   ifelse(C006 < 0, yes=NA, no=C006),
           X001 =   ifelse(X001 < 0, yes=NA, no=X001),
           X003 =   ifelse(X003 < 0, yes=NA, no=X003/10))]
```

Multiple regression

- We conduct the same regression analysis in each `Wave` and `Country`.
- `Wave` is used as a moderator in predicting the estimated regression coefficients (effect sizes).

```
## Function to fit regression model
## y1 to y5: Regression coefficients from A170, A009, A173, C006, X001, and X003.
## v11 to v55: Sampling covariance matrix of the parameter estimates
fun.reg <- function(dt) { fit <- try(lm(A170~A009+A173+C006+X001+X003, data=dt), silent=TRUE)

                          ## If there are errors during the analysis, it returns missing values.
                          if (is.element("try-error", class(fit))) {
                            list(y1=NaN,y2=NaN,y3=NaN,y4=NaN,y5=NaN,
                                 v11=NaN,v21=NaN,v31=NaN,v41=NaN,v51=NaN,
                                 v22=NaN,v32=NaN,v42=NaN,v52=NaN,v33=NaN,
                                 v43=NaN,v53=NaN,v44=NaN,v54=NaN,v55=NaN)
                          } else {
                            ## Extract the regression coefficients excluding the intercept
                            y <- coef(fit)
                            ## Extract the sampling covariance matrix excluding the intercept
                            v <- lav_matrix_vech(vcov(fit)[-1,-1])
                            list(y1=y[2],y2=y[3],y3=y[4],y4=y[5],y5=y[6],
                                 v11=v[1],v21=v[2],v31=v[3],v41=v[4],v51=v[5],
                                 v22=v[6],v32=v[7],v42=v[8],v52=v[9],v33=v[10],
                                 v43=v[11],v53=v[12],v44=v[13],v54=v[14],v55=v[15])
                            }
                          }

########## Split data by Wave and Country and analyze with the fun.reg() function
REM1.reg <- WVS[, fun.reg(.SD), by=list(S002,S003)]

########## Meta-analyze results with a mixed-effects meta-analysis by using "Wave"" as a predictor
REM2.reg <- meta(y=cbind(y1,y2,y3,y4,y5),
                 v=cbind(v11,v21,v31,v41,v51,v22,v32,v42,v52,v33,v43,v53,v44,v54,v55),
                 x=S002, data=REM1.reg,
                 #RE.constraints=Diag(paste(0.1, "*Tau2_", 1:5, "_", 1:5, sep = "")),
                 #RE.lbound=NA,
                 model.name="Regression analysis REM")
## Rerun the analysis to remove error code
## REM2.reg <- rerun(REM2.reg)
```

```r
summary(REM2.reg)
```

```
##
## Call:
## meta(y = cbind(y1, y2, y3, y4, y5), v = cbind(v11, v21, v31,
##     v41, v51, v22, v32, v42, v52, v33, v43, v53, v44, v54, v55),
##     x = S002, data = REM1.reg, model.name = "Regression analysis REM")
##
## 95% confidence intervals: z statistic approximation
## Coefficients:
##               Estimate   Std.Error      lbound      ubound z value
## Intercept1  2.4798e-01  3.6718e-02  1.7601e-01  3.1994e-01  6.7535
## Intercept2  2.4405e-01  1.9082e-02  2.0665e-01  2.8145e-01 12.7898
## Intercept3  4.7955e-01  3.3691e-02  4.1352e-01  5.4559e-01 14.2338
## Intercept4  1.2161e-01  3.4507e-02  5.3975e-02  1.8924e-01  3.5241
## Intercept5  6.0495e-02  1.3172e-02  3.4678e-02  8.6313e-02  4.5926
## Slope1_1    3.4926e-02  7.9917e-03  1.9262e-02  5.0589e-02  4.3703
## Slope2_1   -8.7358e-03  4.1514e-03 -1.6872e-02 -5.9917e-04 -2.1043
## Slope3_1   -2.3119e-02  7.3282e-03 -3.7482e-02 -8.7559e-03 -3.1548
## Slope4_1    1.8811e-03  7.4658e-03 -1.2752e-02  1.6514e-02  0.2520
## Slope5_1   -5.9947e-03  2.8470e-03 -1.1575e-02 -4.1470e-04 -2.1056
## Tau2_1_1    2.1200e-02  2.4216e-03  1.6454e-02  2.5946e-02  8.7546
## Tau2_2_1   -7.7709e-04  8.8281e-04 -2.5074e-03  9.5319e-04 -0.8802
## Tau2_2_2    6.2815e-03  6.4521e-04  5.0170e-03  7.5461e-03  9.7356
## Tau2_3_1   -5.8669e-03  1.6097e-03 -9.0218e-03 -2.7120e-03 -3.6447
## Tau2_3_2   -3.4483e-03  8.4351e-04 -5.1016e-03 -1.7951e-03 -4.0881
## Tau2_3_3    2.0893e-02  2.0290e-03  1.6916e-02  2.4869e-02 10.2971
## Tau2_4_1    9.6175e-04  1.6118e-03 -2.1973e-03  4.1208e-03  0.5967
## Tau2_4_2   -2.9473e-04  8.1345e-04 -1.8891e-03  1.2996e-03 -0.3623
## Tau2_4_3    2.2565e-03  1.4378e-03 -5.6155e-04  5.0746e-03  1.5694
## Tau2_4_4    1.2287e-02  2.1230e-03  8.1257e-03  1.6448e-02  5.7874
## Tau2_5_1    1.5727e-03  6.3030e-04  3.3733e-04  2.8080e-03  2.4952
## Tau2_5_2    8.7684e-05  3.1536e-04 -5.3042e-04  7.0579e-04  0.2780
## Tau2_5_3   -1.6770e-03  5.6525e-04 -2.7849e-03 -5.6918e-04 -2.9669
## Tau2_5_4    1.2997e-04  5.7543e-04 -9.9785e-04  1.2578e-03  0.2259
## Tau2_5_5    2.0429e-03  3.0799e-04  1.4392e-03  2.6465e-03  6.6330
##              Pr(>|z|)
## Intercept1 1.443e-11 ***
## Intercept2 < 2.2e-16 ***
## Intercept3 < 2.2e-16 ***
## Intercept4 0.0004249 ***
## Intercept5 4.379e-06 ***
## Slope1_1   1.241e-05 ***
## Slope2_1   0.0353528 *
## Slope3_1   0.0016061 **
## Slope4_1   0.8010658
## Slope5_1   0.0352368 *
## Tau2_1_1   < 2.2e-16 ***
## Tau2_2_1   0.3787255
## Tau2_2_2   < 2.2e-16 ***
## Tau2_3_1   0.0002677 ***
## Tau2_3_2   4.350e-05 ***
## Tau2_3_3   < 2.2e-16 ***
```

```
## Tau2_4_1   0.5507091
## Tau2_4_2   0.7171115
## Tau2_4_3   0.1165534
## Tau2_4_4   7.149e-09 ***
## Tau2_5_1   0.0125900 *
## Tau2_5_2   0.7809798
## Tau2_5_3   0.0030080 **
## Tau2_5_4   0.8213035
## Tau2_5_5   3.289e-11 ***
## ---
## Signif. codes:  0 '***' 0.001 '**' 0.01 '*' 0.05 '.' 0.1 ' ' 1
##
## Q statistic on the homogeneity of effect sizes: 17711.95
## Degrees of freedom of the Q statistic: 1105
## P value of the Q statistic: 0
##
## Explained variances (R2):
##                              y1        y2        y3        y4     y5
## Tau2 (no predictor)    0.0234364 0.0064104 0.0218444 0.0122459 0.0021
## Tau2 (with predictors) 0.0211999 0.0062815 0.0208926 0.0122867 0.0020
## R2                     0.0954278 0.0200968 0.0435724 0.0000000 0.0271
##
## Number of studies (or clusters): 238
## Number of observed statistics: 1110
## Number of estimated parameters: 25
## Degrees of freedom: 1085
## -2 log likelihood: -1783.684
## OpenMx status1: 0 ("0" or "1": The optimization is considered fine.
## Other values may indicate problems.)
```

Mediation analysis

- A mediation model is fitted by using *satisfaction with your life* (A170), *freedom of choice and control* (A173), and *subjective state of health* (A009) as the dependent variable, the mediator, and the predictor, respectively.
- The following figure displays the mediation model.

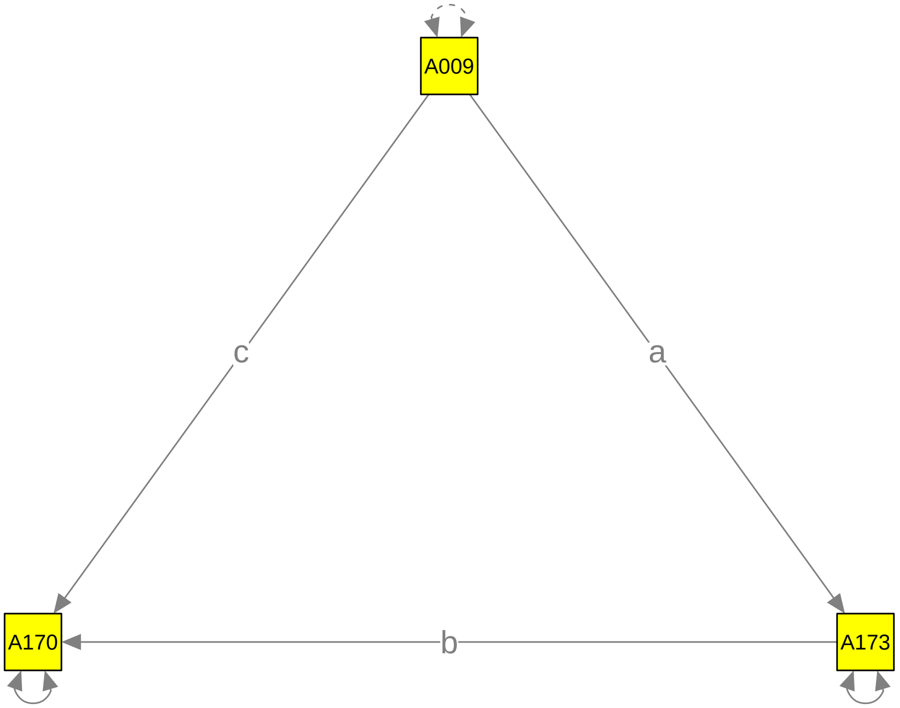


```
## Function to fit a mediation model using sem() function in lavaan,
## where the path coefficients are labelled with "a", "b", and "c."
## y1 and y2: indirect (a*b) and direct effects (c)
## v11, v21, and v22: Sampling covariance matrix of the indirect and direct effects
fun.med <- function(dt) { model.med <- 'A170 ~ b*A173 + c*A009
                                        A173 ~ a*A009
                                        indirect := a*b
                                        direct := c'

                          ## If there are errors during the analysis, it returns missing values.
                          fit <- try(sem(model.med, data=dt), silent=TRUE)

                          if (is.element("try-error", class(fit))) {
                            list(y1=NaN,y2=NaN,v11=NaN,v21=NaN,v22=NaN)
                          } else {
                            ## y: indirect effect and direct effect
                            y <- fit@Model@def.function(.x.=fit@Fit@x)
                            ## x: all parameter estimates
                            x <- fit@Fit@x
                            ## Variance covariance matrix of the parameter estimates
                            VCOV <- vcov(fit)
                            ## Compute the jacobian for 'defined parameters'
                            JAC <- lavaan:::lavJacobianD(func=fit@Model@def.function, x=x)
                            ## Compute the sampling covariance matrix using delta method
                            v <- JAC %*% VCOV %*% t(JAC)
                            list(y1=y[1],y2=y[2],v11=v[1,1],v21=v[2,1],v22=v[2,2]) }}
```

```r
########## Split data by Wave and Country and analyze with the fun.med() function
REM1.med <- WVS[, fun.med(.SD), by=list(S002,S003)]
```

```r
## Show part of the results
head(REM1.med)
```

```
##    S002 S003         y1        y2          v11           v21         v22
## 1:    1   32 0.12712825 0.3943876 0.0014586585 -7.038842e-05 0.005209759
## 2:    1   36 0.07668854 0.2951055 0.0003727852 -3.233129e-05 0.002295134
## 3:    1  246 0.10353528 0.2843115 0.0003172598 -1.008331e-04 0.002114249
## 4:    1  348 0.16762658 0.5094084 0.0006545472 -1.691690e-04 0.004251532
## 5:    1  392 0.11221672 0.3754183 0.0005066221 -2.119145e-04 0.005067255
## 6:    1  410 0.05967650 0.3136188 0.0005241833 -3.915344e-05 0.004806417
```

```r
########## Meta-analyze results with a random-effects meta-analysis
REM2.med <- meta(y=cbind(y1,y2), v=cbind(v11,v21,v22), data=REM1.med,
                 model.name="Mediation analysis REM")

summary(REM2.med)
```

```
##
## Call:
## meta(y = cbind(y1, y2), v = cbind(v11, v21, v22), data = REM1.med,
##     model.name = "Mediation analysis REM")
##
## 95% confidence intervals: z statistic approximation
## Coefficients:
##               Estimate   Std.Error      lbound      ubound z value
## Intercept1  0.13079543  0.00559540  0.11982865  0.14176221 23.3755
## Intercept2  0.56588632  0.01320744  0.54000021  0.59177244 42.8460
## Tau2_1_1    0.00640692  0.00069401  0.00504668  0.00776716  9.2317
## Tau2_2_1    0.00104956  0.00111144 -0.00112881  0.00322794  0.9443
## Tau2_2_2    0.03514025  0.00373117  0.02782728  0.04245321  9.4180
##            Pr(>|z|)
## Intercept1   <2e-16 ***
## Intercept2   <2e-16 ***
## Tau2_1_1     <2e-16 ***
## Tau2_2_1      0.345
## Tau2_2_2     <2e-16 ***
## ---
## Signif. codes:  0 '***' 0.001 '**' 0.01 '*' 0.05 '.' 0.1 ' ' 1
##
## Q statistic on the homogeneity of effect sizes: 6648.819
## Degrees of freedom of the Q statistic: 454
## P value of the Q statistic: 0
##
## Heterogeneity indices (based on the estimated Tau2):
##                               Estimate
## Intercept1: I2 (Q statistic)    0.9574
## Intercept2: I2 (Q statistic)    0.8996
##
## Number of studies (or clusters): 238
```

```
## Number of observed statistics: 456
## Number of estimated parameters: 5
## Degrees of freedom: 451
## -2 log likelihood: -556.4596
## OpenMx status1: 0 ("0" or "1": The optimization is considered fine.
## Other values may indicate problems.)
```

- The following plot shows a multivariate generalization of the average effect size and its 95% confidence interval in univariate meta-analysis.
  - The black dots and the black dashed ellipses are the observed effect sizes and their 95% confidence ellipses in the primary studies.
  - The blue diamond represents the estimated average population effect sizes, while the red ellipse is the 95% confidence ellipse of estimated population average effect sizes.
  - The green ellipse is the 95% confidence ellipse of the random effects. Ninety-five percent of the studies with average population effect sizes falls inside this confidence ellipse in the long run.

```
plot(REM2.med, main="Multivariate meta-analysis",
     axis.label=c("Indirect effect", "Direct effect"),
     study.min.cex=0.6, randeff.ellipse.lty=2,
     randeff.ellipse.lwd=3)
```

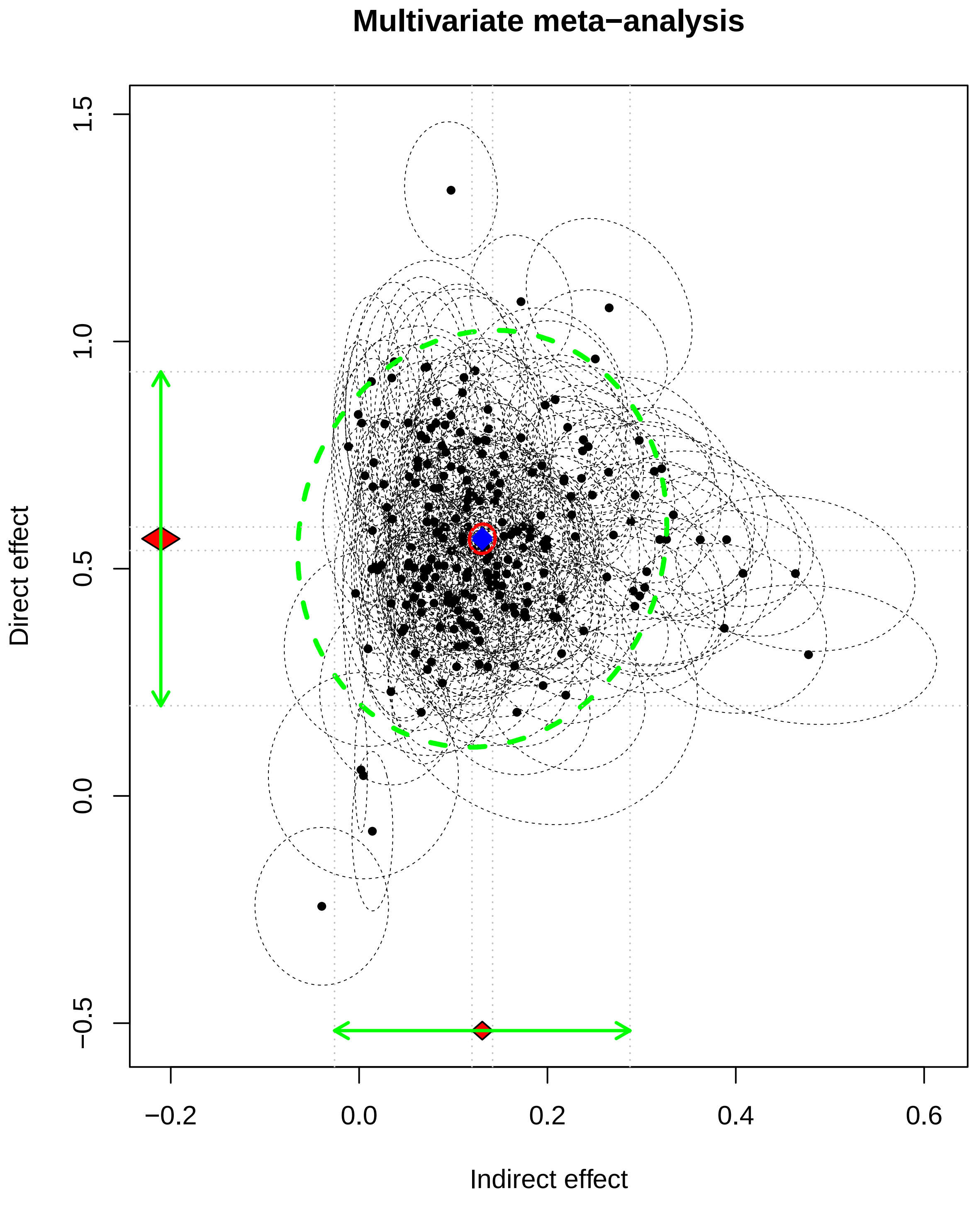


```
########## Meta-analyze results with a mixed-effects meta-analysis
## by using "Wave" (S002) as a predictor
REM3.med <- meta(y=cbind(y1,y2), v=cbind(v11,v21,v22), x=S002, data=REM1.med,
                 model.name="Mediation analysis REM")
```

```r
summary(REM3.med)
```

```
##
## Call:
## meta(y = cbind(y1, y2), v = cbind(v11, v21, v22), x = S002, data = REM1.med,
##     model.name = "Mediation analysis REM")
##
## 95% confidence intervals: z statistic approximation
## Coefficients:
##               Estimate   Std.Error      lbound      ubound z value
## Intercept1  0.14382797  0.01792491  0.10869580  0.17896015  8.0239
## Intercept2  0.37082687  0.04011584  0.29220127  0.44945247  9.2439
## Slope1_1   -0.00301155  0.00392591 -0.01070619  0.00468310 -0.7671
## Slope2_1    0.04505648  0.00880766  0.02779378  0.06231918  5.1156
## Tau2_1_1    0.00636820  0.00069088  0.00501411  0.00772229  9.2176
## Tau2_2_1    0.00132289  0.00105029 -0.00073563  0.00338142  1.2596
## Tau2_2_2    0.03096986  0.00334583  0.02441215  0.03752757  9.2563
##             Pr(>|z|)
## Intercept1 1.110e-15 ***
## Intercept2 < 2.2e-16 ***
## Slope1_1      0.4430
## Slope2_1   3.127e-07 ***
## Tau2_1_1   < 2.2e-16 ***
## Tau2_2_1      0.2078
## Tau2_2_2   < 2.2e-16 ***
## ---
## Signif. codes:  0 '***' 0.001 '**' 0.01 '*' 0.05 '.' 0.1 ' ' 1
##
## Q statistic on the homogeneity of effect sizes: 6648.819
## Degrees of freedom of the Q statistic: 454
## P value of the Q statistic: 0
##
## Explained variances (R2):
##                                y1     y2
## Tau2 (no predictor)    0.0064069 0.0351
## Tau2 (with predictors) 0.0063682 0.0310
## R2                     0.0060435 0.1187
##
## Number of studies (or clusters): 238
## Number of observed statistics: 456
## Number of estimated parameters: 7
## Degrees of freedom: 449
## -2 log likelihood: -582.3821
## OpenMx status1: 0 ("0" or "1": The optimization is considered fine.
## Other values may indicate problems.)
```

Confirmatory factor analysis and reliability generalization: Random-effects models

- The data are grouped according to `Wave` and `Country`.
- Random-effects models are used to account for the differences in `Wave` and `Country`.

- Items used in the analysis:
  - *Justifiable: claiming government benefits to which you are not entitled* (F114)
  - *Justifiable: avoiding a fare on public transport* (F115)
  - *Justifiable: cheating on taxes* (F116)
  - *Justifiable: someone accepting a bribe in the course of their duties* (F117)
  - 1 (Never justifiable) to 10 (Always justifiable); negative values represent missing values. They were recoded into missing values before the analysis.

```
## Clear all objects in the work space
rm(list=ls())

## Load the data
load("WVS.Rdata")

## Select the relevant variables to minimize memory usage
WVS <- WVS[, list(F114, F115, F116, F117, S002, S003)]

## Set Wave and Country as key variables for fast reference
## S002: Wave
## S003: Country
setkeyv(WVS, c("S002", "S003"))

## Recode all negative values as NA
WVS[, `:=`(F114 = ifelse(F114 < 0, yes=NA, no=F114),
           F115 = ifelse(F115 < 0, yes=NA, no=F115),
           F116 = ifelse(F116 < 0, yes=NA, no=F116),
           F117 = ifelse(F117 < 0, yes=NA, no=F117))]
```

Confirmatory factor analysis using the TSSEM approach

- We estimate the correlation matrix in each `Wave` and `Country`.
- The correlation matrices are used to fit a one-factor confirmatory factor analysis with the random-effects two-stage structural equation modeling (TSSEM) approach.

```
## Function to extract correlation matrix and sample sizes
## c21 to c43: Correlation matrix based on pairwise deletion among F114, F115, F116, and F117.
## n: Sample size based on the harmonic mean of the sample sizes in the correlation coefficients.
fun.cor <- function(dt) { ## Calculate the correlation matrix with pairwise deletion
                          fit <- try(suppressWarnings(cor(dt[, 1:4, with=FALSE],
                                     use="pairwise.complete.obs")), silent=TRUE)

                          ## Calculate the sample sizes based on harmonic mean
                          na.n <- t(!is.na(dt[, 1:4, with=FALSE])) %*% !is.na(dt[, 1:4, with=FALSE])
                          pairwise.n <- na.n[lower.tri(na.n)]
                          pairwise.n[pairwise.n==0] <- NA
                          ## harmonic mean
                          n <- as.integer(1/mean(1/pairwise.n, na.rm=TRUE))

                          if (is.element("try-error", class(fit))) {
                            list(c21=NaN,c31=NaN,c41=NaN,c32=NaN,
                                 c42=NaN,c43=NaN,n=NaN)
                          } else {
```

```
                            ## regression coefficients excluding the intercept
                            list(c21=fit[2,1],c31=fit[3,1],c41=fit[4,1],
                                 c32=fit[3,2],c42=fit[4,2],c43=fit[4,3],n=n)
                            }
                          }

########## Split data by Wave and Country and extract the correlation matrices
########## and sample size with the fun.cor() function
stage0.cor <- WVS[, fun.cor(.SD), by=list(S002,S003)]

## Exclude studies without any data
stage0.cor <- stage0.cor[!is.na(n)]

## Show part of the results
head(stage0.cor)
```

```
##    S002 S003       c21       c31       c41       c32       c42       c43
## 1:    1   32 0.4533558 0.3133980 0.2271336 0.4802159 0.3638483 0.2196182
## 2:    1   36 0.5849500 0.3743084 0.4728839 0.5053942 0.4342919 0.3321573
## 3:    1  246 0.3694929 0.2007690 0.1780308 0.4887677 0.2802600 0.3166599
## 4:    1  348 0.2099564        NA 0.2153735        NA 0.2037627        NA
## 5:    1  392 0.4326823 0.3315766 0.3141256 0.6160887 0.4499204 0.4618754
## 6:    1  410 0.3307074 0.2665575 0.2155814 0.3968305 0.2439903 0.4004814
##       n
## 1:  832
## 2: 1201
## 3:  998
## 4: 1407
## 5: 1058
## 6:  909
```

```
## Split the data into a list for ease of data analyses
data.splitted <- split(as.data.frame(stage0.cor), 1:nrow(stage0.cor))

## Convert correlation coefficients into correlation matrices
data.cor <- lapply(data.splitted, function(x) vec2symMat(unlist(x[, 3:8]), diag=FALSE) )

## Extract the sample sizes
data.n <- sapply(data.splitted, function(x) x[, 9])

########## Meta-analyze results with the TSSEM random-effects model
REM1.cfa <- tssem1(data.cor, data.n, method="REM", RE.type="Diag",
                   model.name="One factor model REM")
## Rerun the analysis to remove error code
## REM1.cfa <- rerun(REM1.cfa)
summary(REM1.cfa)
```

```
##
## Call:
## meta(y = ES, v = acovR, RE.constraints = Diag(x = paste(RE.startvalues,
##     "*Tau2_", 1:no.es, "_", 1:no.es, sep = "")), RE.lbound = RE.lbound,
##     I2 = I2, model.name = model.name, suppressWarnings = TRUE,
##     silent = silent, run = run)
```

```
##
## 95% confidence intervals: z statistic approximation
## Coefficients:
##              Estimate Std.Error    lbound    ubound z value  Pr(>|z|)
## Intercept1 0.4304445 0.0086930 0.4134066 0.4474824  49.517 < 2.2e-16 ***
## Intercept2 0.3707085 0.0090174 0.3530347 0.3883823  41.110 < 2.2e-16 ***
## Intercept3 0.3220982 0.0095944 0.3032935 0.3409029  33.571 < 2.2e-16 ***
## Intercept4 0.4796892 0.0083280 0.4633665 0.4960118  57.599 < 2.2e-16 ***
## Intercept5 0.3804907 0.0088057 0.3632317 0.3977496  43.209 < 2.2e-16 ***
## Intercept6 0.4987723 0.0105300 0.4781339 0.5194106  47.367 < 2.2e-16 ***
## Tau2_1_1   0.0162257 0.0015820 0.0131251 0.0193263  10.257 < 2.2e-16 ***
## Tau2_2_2   0.0172790 0.0016984 0.0139503 0.0206077  10.174 < 2.2e-16 ***
## Tau2_3_3   0.0203306 0.0019599 0.0164894 0.0241719  10.373 < 2.2e-16 ***
## Tau2_4_4   0.0146320 0.0014426 0.0118046 0.0174594  10.143 < 2.2e-16 ***
## Tau2_5_5   0.0167704 0.0016270 0.0135816 0.0199591  10.308 < 2.2e-16 ***
## Tau2_6_6   0.0241432 0.0023285 0.0195794 0.0287071  10.368 < 2.2e-16 ***
## ---
## Signif. codes:  0 '***' 0.001 '**' 0.01 '*' 0.05 '.' 0.1 ' ' 1
##
## Q statistic on the homogeneity of effect sizes: 74439.54
## Degrees of freedom of the Q statistic: 1328
## P value of the Q statistic: 0
##
## Heterogeneity indices (based on the estimated Tau2):
##                              Estimate
## Intercept1: I2 (Q statistic)   0.9765
## Intercept2: I2 (Q statistic)   0.9731
## Intercept3: I2 (Q statistic)   0.9759
## Intercept4: I2 (Q statistic)   0.9749
## Intercept5: I2 (Q statistic)   0.9754
## Intercept6: I2 (Q statistic)   0.9884
##
## Number of studies (or clusters): 230
## Number of observed statistics: 1334
## Number of estimated parameters: 12
## Degrees of freedom: 1322
## -2 log likelihood: -1509.782
## OpenMx status1: 0 ("0" or "1": The optimization is considered fine.
## Other values may indicate problems.)
```

```
## Show the pooled correlation matrix
vec2symMat(coef(REM1.cfa, select="fixed"), diag=FALSE)
```

```
##           [,1]      [,2]      [,3]      [,4]
## [1,] 1.0000000 0.4304445 0.3707085 0.3220982
## [2,] 0.4304445 1.0000000 0.4796892 0.3804907
## [3,] 0.3707085 0.4796892 1.0000000 0.4987723
## [4,] 0.3220982 0.3804907 0.4987723 1.0000000
```

```
## Show the variance components of the random effects
Diag(coef(REM1.cfa, select="random"))
```

```
##           [,1]       [,2]       [,3]       [,4]       [,5]       [,6]
```

```
## [1,] 0.0162257 0.00000000 0.00000000 0.00000000 0.00000000 0.00000000
## [2,] 0.0000000 0.01727902 0.00000000 0.00000000 0.00000000 0.00000000
## [3,] 0.0000000 0.00000000 0.02033064 0.00000000 0.00000000 0.00000000
## [4,] 0.0000000 0.00000000 0.00000000 0.01463205 0.00000000 0.00000000
## [5,] 0.0000000 0.00000000 0.00000000 0.00000000 0.01677037 0.00000000
## [6,] 0.0000000 0.00000000 0.00000000 0.00000000 0.00000000 0.02414322
```

```r
## Setup a one-factor CFA model in RAM specification
A1 <- matrix(c("0.2*F114", "0.2*F115", "0.2*F116", "0.2*F117",0), ncol=1)
A1 <- cbind(matrix(0, ncol=4, nrow=5), A1)
dimnames(A1)[[1]] <- dimnames(A1)[[2]] <- c("F114","F115","F116","F117","Fraud")

## A matrix for regression coefficients and factor loadings
A1
```

```
##       F114 F115 F116 F117 Fraud
## F114  "0"  "0"  "0"  "0"  "0.2*F114"
## F115  "0"  "0"  "0"  "0"  "0.2*F115"
## F116  "0"  "0"  "0"  "0"  "0.2*F116"
## F117  "0"  "0"  "0"  "0"  "0.2*F117"
## Fraud "0"  "0"  "0"  "0"  "0"
```

```r
S1 <- Diag(c("0.2*ErrVar_F114", "0.2*ErrVar_F115",
             "0.2*ErrVar_F116", "0.2*ErrVar_F117", "1") )
dimnames(S1)[[1]] <- dimnames(S1)[[2]] <- c("F114","F115","F116","F117","Fraud")

## S matrix for variances and covariances
S1
```

```
##       F114              F115              F116
## F114  "0.2*ErrVar_F114" "0"               "0"
## F115  "0"               "0.2*ErrVar_F115" "0"
## F116  "0"               "0"               "0.2*ErrVar_F116"
## F117  "0"               "0"               "0"
## Fraud "0"               "0"               "0"
##       F117              Fraud
## F114  "0"               "0"
## F115  "0"               "0"
## F116  "0"               "0"
## F117  "0.2*ErrVar_F117" "0"
## Fraud "0"               "1"
```

```r
F1 <- create.Fmatrix(c(1,1,1,1,0), as.mxMatrix=FALSE)
dimnames(F1)[[1]] <- c("F114","F115","F116","F117")
dimnames(F1)[[2]] <- c("F114","F115","F116","F117","Fraud")

## F matrix to select observed variables
F1
```

```
##      F114 F115 F116 F117 Fraud
## F114    1    0    0    0     0
## F115    0    1    0    0     0
## F116    0    0    1    0     0
## F117    0    0    0    1     0
```

```r
########## Fit a one-factor CFA model on the average correlation matrix
REM2.cfa <- tssem2(REM1.cfa, Amatrix=A1, Smatrix=S1, Fmatrix=F1, diag.constraints=TRUE,
                   intervals.type="LB", model.name="One factor model REM Stage 2 analysis")
summary(REM2.cfa)
```

```
##
## Call:
## wls(Cov = pooledS, asyCov = asyCov, n = tssem1.obj$total.n, Amatrix = Amatrix,
##     Smatrix = Smatrix, Fmatrix = Fmatrix, diag.constraints = diag.constraints,
##     cor.analysis = cor.analysis, intervals.type = intervals.type,
##     mx.algebras = mx.algebras, model.name = model.name, suppressWarnings = suppressWarnings,
##     silent = silent, run = run)
##
## 95% confidence intervals: Likelihood-based statistic
## Coefficients:
##             Estimate Std.Error  lbound  ubound z value Pr(>|z|)
## F114         0.56264        NA 0.54597 0.57945      NA       NA
## F115         0.68028        NA 0.66209 0.69875      NA       NA
## F116         0.72028        NA 0.70081 0.74006      NA       NA
## F117         0.60689        NA 0.58881 0.62514      NA       NA
## ErrVar_F114  0.68344        NA 0.66423 0.70193      NA       NA
## ErrVar_F115  0.53722        NA 0.51175 0.56160      NA       NA
## ErrVar_F116  0.48119        NA 0.45232 0.50888      NA       NA
## ErrVar_F117  0.63168        NA 0.60922 0.65333      NA       NA
##
## Goodness-of-fit indices:
##                                                  Value
## Sample size                                 3.1200e+05
## Chi-square of target model                  9.9253e+01
## DF of target model                          2.0000e+00
## p value of target model                     0.0000e+00
## Number of constraints imposed on "Smatrix" 4.0000e+00
## DF manually adjusted                        0.0000e+00
## Chi-square of independence model            1.2203e+04
## DF of independence model                    6.0000e+00
## RMSEA                                       1.2500e-02
## RMSEA lower 95% CI                          1.0500e-02
## RMSEA upper 95% CI                          1.4600e-02
## SRMR                                        3.8300e-02
## TLI                                         9.7610e-01
## CFI                                         9.9200e-01
## AIC                                         9.5253e+01
## BIC                                         7.3951e+01
## OpenMx status1: 0 ("0" or "1": The optimization is considered fine.
## Other values indicate problems.)
```

```r
## Convert the model to semPlotModel object
library("semPlot")
my.plot <- meta2semPlot(REM2.cfa, manNames=c("F114","F115","F116","F117"),
                        latNames=c("Fraud"))

## Plot the model with labels
semPaths(my.plot, whatLabels="est", nCharEdges=10, nCharNodes=10,
```

```
        edge.label.cex=1.3, color="yellow")
```

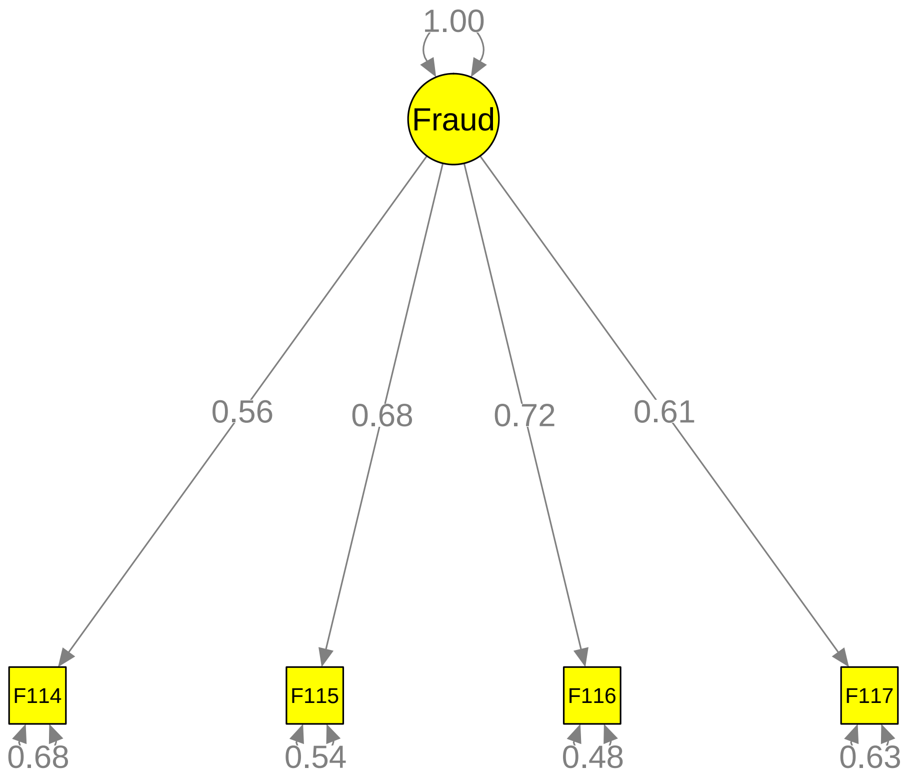

Reliability generalizability with a random-effects model

- The coefficient alpha and its sampling variance are estimated in each `Wave` and `Country`.
- Random- and mixed-effects meta-analyses are tested.

```
## Function to extract coefficient alpha and its sampling variance
## y: estimated coefficient alpha
## v: sampling variance of coefficient alpha
fun.rel <- function(dt) { Cov <- try(cov(dt[, 1:4, with=FALSE],
                                          use="pairwise.complete.obs"), silent=TRUE)
                          na.n <- t(!is.na(dt[, 1:4, with=FALSE])) %*% !is.na(dt[, 1:4, with=FALSE])
                          pairwise.n <- na.n[lower.tri(na.n, diag=TRUE)]
                          pairwise.n[pairwise.n==0] <- NA
                          ## harmonic mean
                          n <- as.integer(1/mean(1/pairwise.n, na.rm=TRUE))

                          if (is.element("try-error", class(Cov))) {
                            list(y=NaN,v=NaN)
                          } else {
                            if (any(is.na(Cov))) {
                              list(y=NaN,v=NaN)
                            } else {
```

```r
                              ## no. of items
                              q <- ncol(Cov)
                              var.item <- sum(diag(Cov))
                              var.scale <- sum(Cov)
                              ## y: coefficient alpha
                              y <- q*(1-var.item/var.scale)/(q-1)
                              ## Bonett (2010, Eq.5)
                              ## v: sampling variance of y (Bonett, 2010, Eq. 5)
                              v <- 2*q*(1-y)^2/((q-1)*(n-2))
                              list(y=y,v=v)
                            }
                          }
                        }

########## Split data by Wave and Country and analyze data with the fun.rel() function
REM1.rel <- WVS[, fun.rel(.SD), by=list(S002,S003)]

## Adjust the scale so that Wave 1 is S002=0.
REM1.rel[, `:=`(S002 = S002-1)]

########## Meta-analyze results with a random-effects meta-analysis by using "Wave"" as a predictor
REM2.rel <- meta(y=y, v=v, x=S002, data=REM1.rel,
                 model.name="Reliability generalization REM")
summary(REM2.rel)
```

```
##
## Call:
## meta(y = y, v = v, x = S002, data = REM1.rel, model.name = "Reliability generalization REM")
##
## 95% confidence intervals: z statistic approximation
## Coefficients:
##              Estimate  Std.Error     lbound     ubound z value  Pr(>|z|)
## Intercept1 0.63529896 0.01655030 0.60286098 0.66773695 38.3860 < 2.2e-16
## Slope1_1   0.02123623 0.00457418 0.01227101 0.03020145  4.6426  3.44e-06
## Tau2_1_1   0.00871555 0.00086861 0.00701311 0.01041799 10.0339 < 2.2e-16
##
## Intercept1 ***
## Slope1_1   ***
## Tau2_1_1   ***
## ---
## Signif. codes:  0 '***' 0.001 '**' 0.01 '*' 0.05 '.' 0.1 ' ' 1
##
## Q statistic on the homogeneity of effect sizes: 24774.77
## Degrees of freedom of the Q statistic: 216
## P value of the Q statistic: 0
##
## Explained variances (R2):
##                           y1
## Tau2 (no predictor)    0.0096
## Tau2 (with predictors) 0.0087
## R2                     0.0926
##
## Number of studies (or clusters): 238
```

```
## Number of observed statistics: 217
## Number of estimated parameters: 3
## Degrees of freedom: 214
## -2 log likelihood: -404.9844
## OpenMx status1: 0 ("0" or "1": The optimization is considered fine.
## Other values may indicate problems.)
```

Settings of the R system

```
sessionInfo()
```

```
## R version 3.2.5 (2016-04-14)
## Platform: x86_64-w64-mingw32/x64 (64-bit)
## Running under: Windows 7 x64 (build 7601) Service Pack 1
##
## locale:
## [1] LC_COLLATE=English_United States.1252
## [2] LC_CTYPE=English_United States.1252
## [3] LC_MONETARY=English_United States.1252
## [4] LC_NUMERIC=C
## [5] LC_TIME=English_United States.1252
##
## attached base packages:
## [1] parallel  stats     graphics  grDevices utils     datasets  methods
## [8] base
##
## other attached packages:
## [1] metaSEM_0.9.8    OpenMx_2.5.2     Rcpp_0.12.4      Matrix_1.2-6
## [5] MASS_7.3-45      digest_0.6.9     data.table_1.9.6 lavaan_0.5-20
## [9] semPlot_1.0.1
##
## loaded via a namespace (and not attached):
##  [1] splines_3.2.5       ellipse_0.3-8       gtools_3.5.0
##  [4] Formula_1.2-1       stats4_3.2.5        latticeExtra_0.6-28
##  [7] yaml_2.1.13         d3Network_0.5.2.1   lisrelToR_0.1.4
## [10] pbivnorm_0.6.0      lattice_0.20-33     quantreg_5.21
## [13] quadprog_1.5-5      chron_2.3-47        RColorBrewer_1.1-2
## [16] ggm_2.3             minqa_1.2.4         colorspace_1.2-6
## [19] htmltools_0.3.5     plyr_1.8.3          psych_1.6.4
## [22] XML_3.98-1.4        SparseM_1.7         corpcor_1.6.8
## [25] scales_0.4.0        glasso_1.8          sna_2.3-2
## [28] whisker_0.3-2       jpeg_0.1-8          fdrtool_1.2.15
## [31] lme4_1.1-12         MatrixModels_0.4-1  huge_1.2.7
## [34] arm_1.8-6           rockchalk_1.8.101   mgcv_1.8-12
## [37] car_2.1-2           ggplot2_2.1.0       nnet_7.3-12
## [40] pbkrtest_0.4-6      mnormt_1.5-4        survival_2.39-3
## [43] magrittr_1.5        evaluate_0.9        nlme_3.1-127
## [46] foreign_0.8-66      tools_3.2.5         formatR_1.4
## [49] stringr_1.0.0       munsell_0.4.3       cluster_2.0.4
## [52] sem_3.1-7           grid_3.2.5          nloptr_1.0.4
## [55] rjson_0.2.15        igraph_1.0.1        rmarkdown_0.9.6
```

```
## [58] boot_1.3-18        mi_1.0              gtable_0.2.0
## [61] abind_1.4-3        reshape2_1.4.1      qgraph_1.3.3
## [64] gridExtra_2.2.1    knitr_1.13          Hmisc_3.17-4
## [67] stringi_1.0-1      matrixcalc_1.0-3    rpart_4.1-10
## [70] acepack_1.3-3.3    png_0.1-7           coda_0.18-1
```
