# Supplementary material for: Analyzing Big Data in Psychology: A Split/Analyze/Meta-Analyze Approach
Source: Front Psychol. 2016 May 23;7:738. doi: 10.3389/fpsyg.2016.00738 (PMC4876837; doi:10.3389/fpsyg.2016.00738)
Supplement: Supplementary file 2 [file DataSheet2.pdf]

Illustrations with the Airlines data in R

Mike Cheung and Suzanne Jak

12 May 2016

Contents

Introduction **1**

Data preparation **1**

Installing the R packages . . . 2
Preparing the datasets . . . 2
Descriptive statistics . . . 4
Read the database into R . . . 4
Display the summary and figures . . . 5

Regression analysis **9**

Read and process data from the database . . . 9
Conducting a multivariate random and mixed-effects meta-analysis . . . 11

Mixed-effects model **14**

Read and process data from the database . . . 14
Conducting a multivariate random-effects meta-analysis . . . 15
Conducting a multivariate mixed-effects meta-analysis . . . 17

Settings of the R system **19**

Introduction

- This is an appendix in Cheung and Jak (2016).
- Cheung, M. W.-L., & Jak, S. (2016). Analyzing big data in psychology: A split/analyze/meta-analyze approach. *Frontiers in Psychology*, *7*(738). http://doi.org/10.3389/fpsyg.2016.00738
- The updated version is available at https://github.com/mikewlcheung/code-in-articles/tree/master/Cheung%20and%20Jak%202016/.

Data preparation

- Before running the analyses, we need to install some R packages and download the data. The analyses should run fine in computer systems with at least 8GB RAM.

Installing the R packages

- R can be downloaded at http://www.r-project.org/.
- We only need to install them once.

```
## Installing R packages from the CRAN
install.packages(c("RSQLite", "dplyr", "lme4", "R.utils", "metaSEM"))
```

Preparing the datasets

- The datasets include more than 123 million records on 29 variables.
- The datasets are available at http://stat-computing.org/dataexpo/2009/the-data.html.
- The following R code is used to download the compressed files and uncompress them in the local harddisk.
- The compressed data sets are 1.7 GB in size, while the uncompressed files are 12 GB in size.
- Please make sure that there is enough space to store the files. Moreover, it may take a long time to download the files and uncompress them.

```
library("R.utils")

## Years of the data
years <- 1987:2008

## Create http addresses for download
http.names <- paste("http://stat-computing.org/dataexpo/2009/",
                    years, ".csv.bz2", sep="")

## Show the first few items
head(http.names)
```

```
## [1] "http://stat-computing.org/dataexpo/2009/1987.csv.bz2"
## [2] "http://stat-computing.org/dataexpo/2009/1988.csv.bz2"
## [3] "http://stat-computing.org/dataexpo/2009/1989.csv.bz2"
## [4] "http://stat-computing.org/dataexpo/2009/1990.csv.bz2"
## [5] "http://stat-computing.org/dataexpo/2009/1991.csv.bz2"
## [6] "http://stat-computing.org/dataexpo/2009/1992.csv.bz2"
```

```
## Create file names to save in the local harddisk
file.names <- paste(years, ".csv.bz2", sep="")

## Show the first few items
head(file.names)
```

```
## [1] "1987.csv.bz2" "1988.csv.bz2" "1989.csv.bz2" "1990.csv.bz2"
## [5] "1991.csv.bz2" "1992.csv.bz2"
```

```
## Download the files
## This may take a while depending on the internet connectivity.
for (i in 1:length(http.names)) {
  download.file(http.names[i], file.names[i])
}
```

```r
## Uncompress the files
## remove=FALSE: not to remove the compressed files
for (i in 1:length(file.names)) {
  bunzip2(file.names[i], overwrite=TRUE, remove=FALSE)
  cat("Completed file: ", file.names[i], "\n")
}
```

- Since most big data sets are stored in database format, we convert the downloaded data sets into a database. This illustrates how the proposed split-analyze-combine model can be applied to realistic environments.
- After running the following R code, a SQLite database called `1987_2008.sqlite`, which is about 14.3 GB in size, will be created. The following analyses are based on this file.

```r
library("RSQLite")

## Set up a connection and create empty database in the working directory
## Name of the dBase: 1987_2008.sqlite
db <- dbConnect(SQLite(), dbname="1987_2008.sqlite")

## Create empty table 'ontime' in database, define variable names and type of fields
## int = integer, varachar(n) = string of length n
## The full list of variable names is available at
## http://stat-computing.org/dataexpo/2009/the-data.html
dbSendQuery(conn = db,
"create table ontime (
  Year int,
  Month int,
  DayofMonth int,
  DayOfWeek int,
  DepTime int,
  CRSDepTime int,
  ArrTime int,
  CRSArrTime int,
  UniqueCarrier varchar(5),
  FlightNum int,
  TailNum varchar(8),
  ActualElapsedTime int,
  CRSElapsedTime int,
  AirTime int,
  ArrDelay int,
  DepDelay int,
  Origin varchar(3),
  Dest varchar(3),
  Distance int,
  TaxiIn int,
  TaxiOut int,
  Cancelled int,
  CancellationCode varchar(1),
  Diverted varchar(1),
  CarrierDelay int,
  WeatherDelay int,
  NASDelay int,
  SecurityDelay int,
```

```
  LateAircraftDelay int
)")

## Create a vector with the names of the .csv files (files are in the working directory)
datasets <- paste(1987:2008, ".csv", sep="")

## Putting the data in the database
## for the first dataset, the header is read in, for the following,
## the first line (header) is skipped
## It took about 16 minutes in our computer.
for (i in 1:length(datasets)){
  if (i == 1) {
    dbWriteTable(conn = db, name = "ontime", value = datasets[i],
                 row.names = FALSE, header = TRUE, append = TRUE)
   } else {
        dbWriteTable(conn = db, name = "ontime", value = datasets[i],
                 row.names = FALSE, header = FALSE, append = TRUE, skip = 1) }
    cat("Completed dataset: ", datasets[i], "\n")
}

## Create indexes for year and Origin, which speeds up later analysis
## It took about 15 minutes in our computer.
dbGetQuery(conn=db, 'create index year on ontime(year)')
dbGetQuery(conn=db, 'create index Origin on ontime(Origin)')

## Show the tables that are in the database
dbListTables(db)

## Show the variables in the table
dbListFields(db, "ontime")

## Close connection to the database
dbDisconnect(db)
```

Descriptive statistics

- We first demonstrate how to obtain some descriptive statistics before conducting inferential statistics.

Read the database into R

- Since it takes some time to process large data, we will read the database file only if the R image `airlines1.Rdata`, which has been saved before, is not available. In general, it is a good idea to save the processed data for further analyses.
- We summarize the means of the arrival delay, departure delay, and distance between airports per year and month.

```
## Library to read SQLite data and process the data
library("dplyr")

## Read the SQLite data only if the R image is not available
if (!file.exists("airlines1.Rdata")) {
```

```
  ## Read the SQLite data
  my.db <- src_sqlite("1987_2008.sqlite")

  ## Read the table "ontime" into "my.df"
  my.df <- tbl(my.db, "ontime")

  ## Calculate the means of ArrDelay, DepDelay, and total no. of flights
  ## by year and month
  my.summary <- my.df %>%
    group_by(Year, Month) %>%
    summarise(arr_delay=mean(ArrDelay),
              dep_delay=mean(DepDelay),
              distance=mean(Distance),
              flights=n())

  ## Convert it into data.frame to avoid rerunning it again
  my.summary <- data.frame(my.summary)

  ## Sort it by Year and Month
  my.summary <- arrange(my.summary, Year, as.numeric(Month))

  ## Save it to avoid rerunning it again
  save(my.summary, file="airlines1.Rdata")
}
```

Display the summary and figures

- The red lines in the figures refer to the *September 11 attacks*.

```
## Load the summary from R image
load("airlines1.Rdata")

## Display the first few cases of the aggregated means
head(my.summary)
```

```
##   Year Month arr_delay dep_delay distance flights
## 1 1987    10  5.953324  5.023688 587.5488  448620
## 2 1987    11  8.341280  7.040165 590.3340  422803
## 3 1987    12 13.562419 11.782452 594.5194  440403
## 4 1988     1 11.559780 10.231040 594.1728  436950
## 5 1988     2  9.190616  8.214260 596.5373  412579
## 6 1988     3  7.019603  6.710859 601.2047  445080
```

```
## Display the last few cases of the aggregated means
tail(my.summary)
```

```
##     Year Month  arr_delay dep_delay distance flights
## 250 2008     7  9.7763528 11.608261 739.2582  627931
## 251 2008     8  6.7807846  9.460311 737.9908  612279
## 252 2008     9  0.6840184  3.889212 712.6817  540908
## 253 2008    10  0.4124073  3.782569 712.5816  556205
```

```
## 254 2008    11  1.9954441  5.376890 717.7072  523272
## 255 2008    12 16.0618708 16.763552 723.2746  544958
```

```r
## values for x axis
x <- 1:nrow(my.summary)

## Plot the no. of flights
plot(x, my.summary$flights, type="l", xaxt="n",
     xlab="Year", ylab="Numbers of flights per month",
     main="Numbers of flights per month by years (1987-2008)")
abline(v=c(x[my.summary$Month=="1"],256), lty=2)
abline(v=168, lwd=3, col="red")
axis(1, at=c(-3, x[my.summary$Month=="6"]), labels=1987:2008)
```

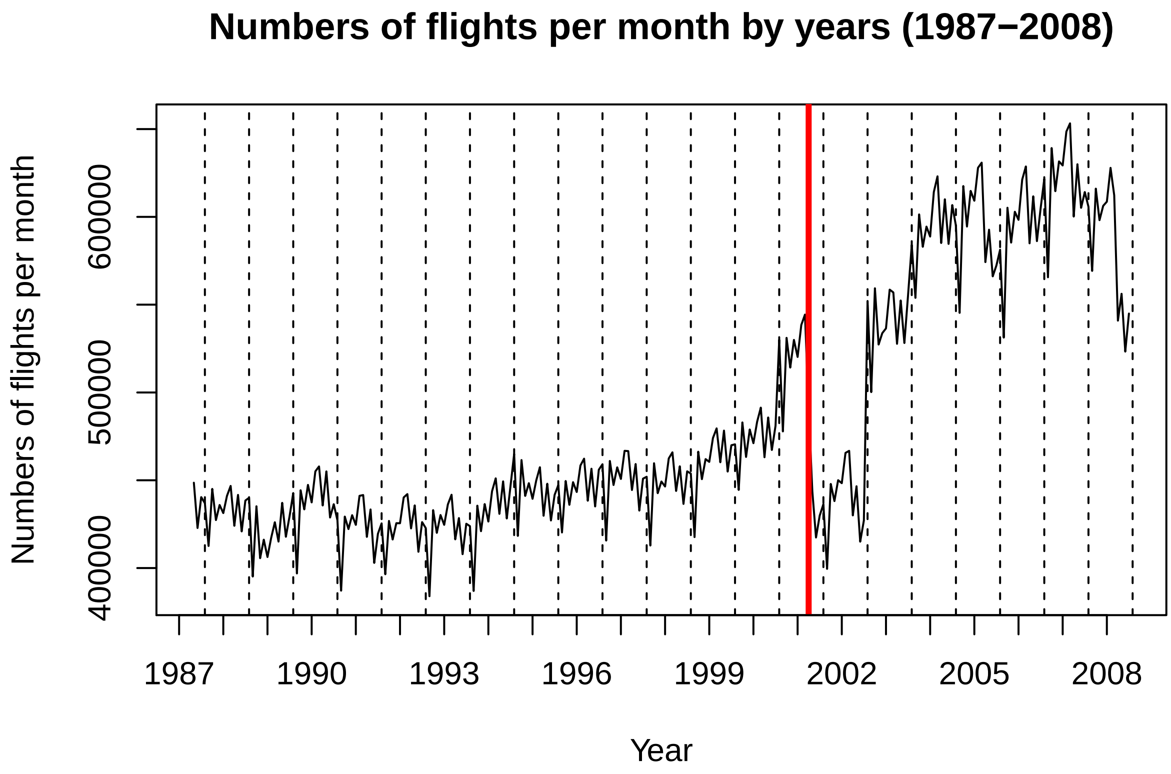


```r
## Plot the delay time
par(mfrow=c(3,1))
plot(x, my.summary$arr_delay, type="l", xaxt="n",
     xlab="Year", ylab="Arrival delay (min)",
     main="Arrival delay by years and months")
abline(v=c(x[my.summary$Month=="1"],256), lty=2)
abline(v=168, lwd=3, col="red")
axis(1, at=c(-3, x[my.summary$Month=="6"]), labels=1987:2008)

plot(x, my.summary$dep_delay, type="l", xaxt="n",
     xlab="Year", ylab="Departure delay (min)",
```

```
     main="Departure delay by years and months")
abline(v=c(x[my.summary$Month=="1"],256), lty=2)
abline(v=168, lwd=3, col="red")
axis(1, at=c(-3, x[my.summary$Month=="6"]), labels=1987:2008)

plot(x, with(my.summary, arr_delay-dep_delay), type="l", xaxt="n",
     xlab="Year", ylab="Departure delay (min)",
     main="Arrival minus departure delay by years and months")
abline(v=c(x[my.summary$Month=="1"],256), lty=2)
abline(v=168, lwd=3, col="red")
abline(h=0, lty=2)
axis(1, at=c(-3, x[my.summary$Month=="6"]), labels=1987:2008)
```

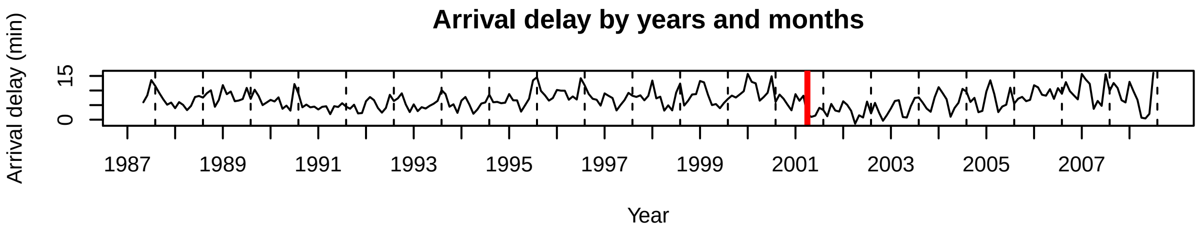

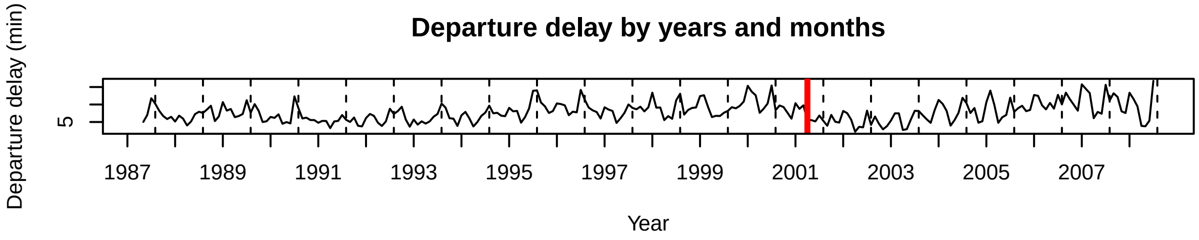

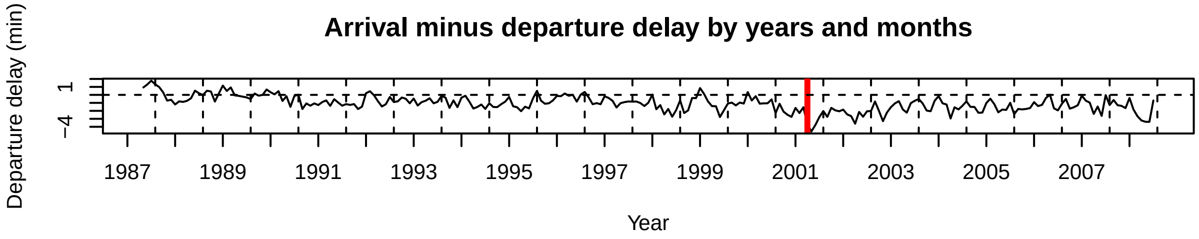


```
## Plot the scatter plot
panel.cor <- function(x, y, digits = 2, prefix = "", cex.cor=2, ...)
{
    usr <- par("usr"); on.exit(par(usr))
    par(usr = c(0, 1, 0, 1))
    r <- cor(x, y)
    txt <- format(c(r, 0.123456789), digits = digits)[1]
    txt <- paste0(prefix, txt)
    text(0.5, 0.5, txt, cex = cex.cor)
}

panel.hist <- function(x, ...)
```

```
{
    usr <- par("usr"); on.exit(par(usr))
    par(usr = c(usr[1:2], 0, 1.5) )
    h <- hist(x, plot = FALSE)
    breaks <- h$breaks; nB <- length(breaks)
    y <- h$counts; y <- y/max(y)
    rect(breaks[-nB], 0, breaks[-1], y, col = "grey", ...)
}

pairs(my.summary[, c("arr_delay", "dep_delay", "distance")],
      lower.panel = panel.smooth, upper.panel = panel.cor,
      diag.panel = panel.hist)
```

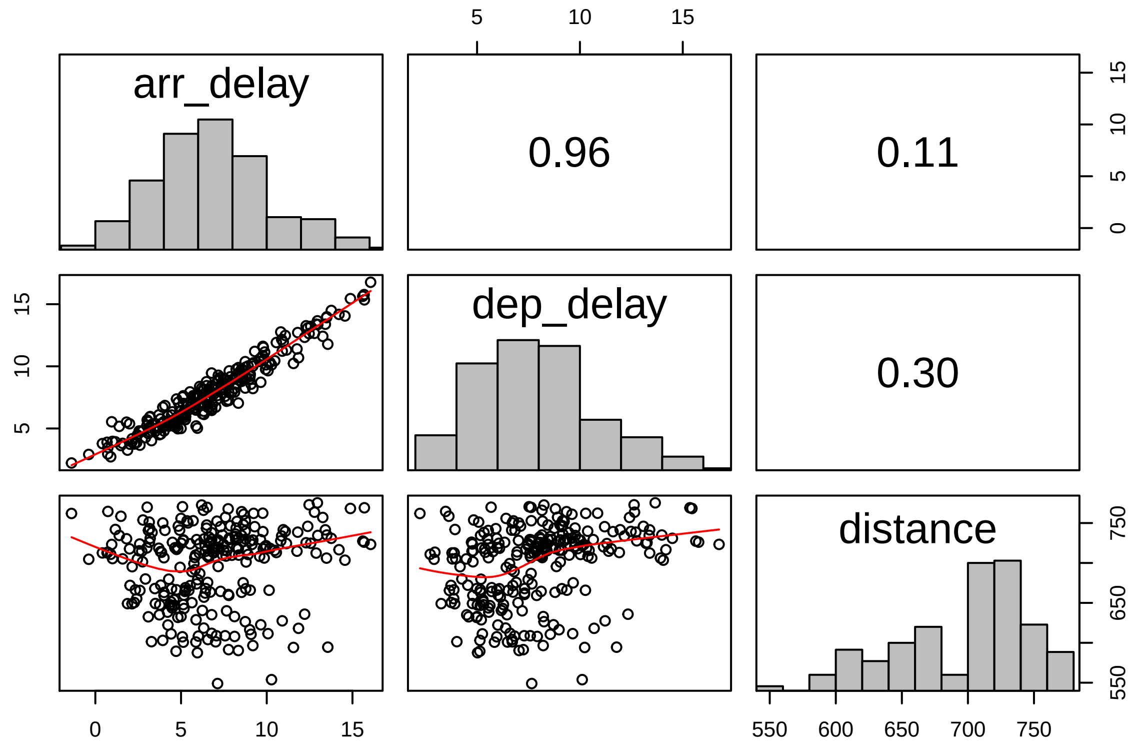


```
## Ecological analysis: Regression analysis on the aggregated means
## I(distance/1000): Distance is divided by 1000 to improve numerical stability.
summary( lm(arr_delay~dep_delay+I(distance/1000), data=my.summary) )
```

```
##
## Call:
## lm(formula = arr_delay ~ dep_delay + I(distance/1000), data = my.summary)
##
## Residuals:
##      Min       1Q   Median       3Q      Max
## -2.67649 -0.37495 -0.03299  0.49619  1.77611
##
```

```
## Coefficients:
##                    Estimate Std. Error t value Pr(>|t|)    
## (Intercept)         6.45746    0.58574   11.03   <2e-16 ***
## dep_delay           1.20842    0.01535   78.73   <2e-16 ***
## I(distance/1000) -13.16094    0.87376  -15.06   <2e-16 ***
## ---
## Signif. codes:  0 '***' 0.001 '**' 0.01 '*' 0.05 '.' 0.1 ' ' 1
##
## Residual standard error: 0.6588 on 252 degrees of freedom
## Multiple R-squared:  0.9614, Adjusted R-squared:  0.9611
## F-statistic:  3141 on 2 and 252 DF,  p-value: < 2.2e-16
```

Regression analysis

- We regress `ArrDelay` on `DepDelay` and `Distance` on each year.
- The following figure displays the regression model.

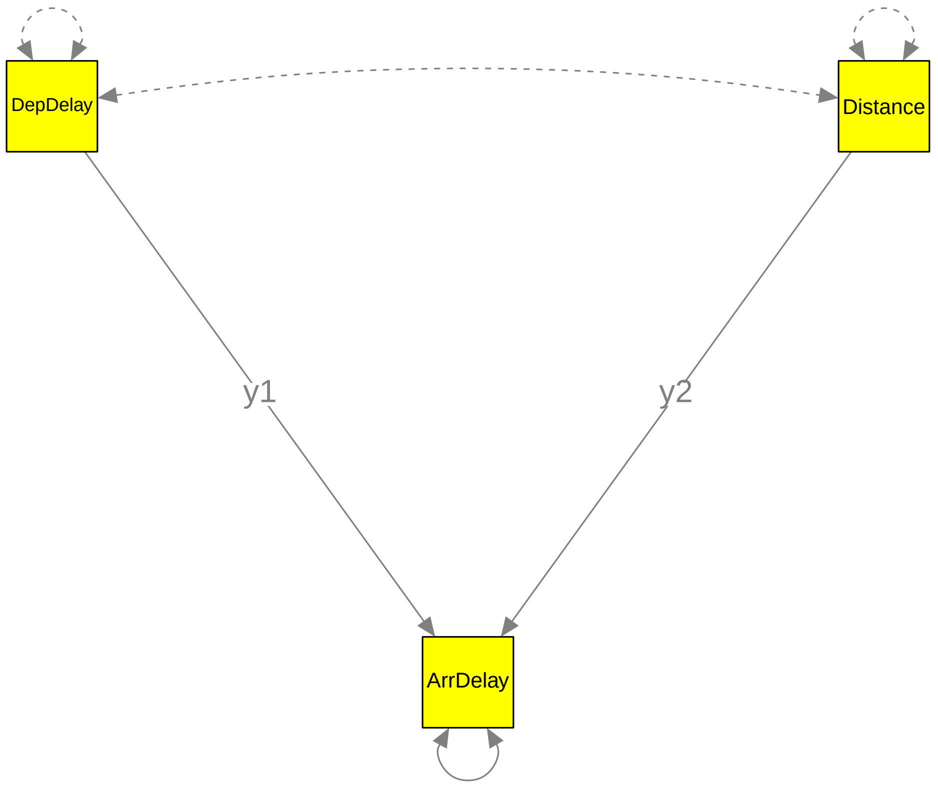

Read and process data from the database

```
library("dplyr")

## Read the SQLite data only if the R image is not available
```

```r
if (!file.exists("airlines2.Rdata")) {

  ## Function to fit regression analysis
  ## I(Distance/1000): Distance is divided by 1000 to improve numerical stability.
  ## y1 and y2: Regression coefficients from Distance and DepDelay.
  ## v11 to v22: Sampling covariance matrix of the parameter estimates
  fun.reg <- function(dt) { fit <- try(lm(ArrDelay~DepDelay+I(Distance/1000), data=dt), silent=TRUE)

                          if (is.element("try-error", class(fit))) {
                            list(y1=NaN,y2=NaN,
                                 v11=NaN,v21=NaN,v22=NaN)
                          } else {
                            ## regression coefficients excluding the intercept
                            y <- coef(fit)
                            ## sampling variance covariance matrix excluding the intercept
                            v <- vech(vcov(fit)[-1,-1])
                            list(y1=y[2],y2=y[3],
                                 v11=v[1],v21=v[2],v22=v[3])}}

  ## Connect to database
  my.db <- src_sqlite("1987_2008.sqlite", create = FALSE)

  ## Connect to table
  my.df <- tbl(my.db, "ontime")

  ## Analyze the data per year

  ## Data.frame to store output
  meta.df <- data.frame(year=NA,y1=NA,y2=NA,v11=NA,v21=NA,v22=NA)

  years <- 1987:2008

  ## It took about 9 minutes in our computer
  for (i in 1:length(years)){

    ## Select year and variables
    c0 <- filter(my.df, Year==years[i])
    c1 <- dplyr::select(c0, ArrDelay, DepDelay, Distance)
    ## Pull data into R
    data <- collect(c1)

    ## Fit regression model and store results
    meta.df[i,] <- c(years[i], unlist(fun.reg(data)))

    ## Clear memory
    rm(data)
    cat("Completed year: ", years[i], "\n")
  }

  ## Save the data for further analyses
  save(meta.df, file = "airlines2.RData")
}
```

Conducting a multivariate random and mixed-effects meta-analysis

- The regression coefficients on `DepDelay` (y1) and on `Distance` (y2) are considered as multiple effect sizes.
- Random-effects multivariate meta-analysis is conducted to account for the differences in `year`. Moreover, `year` is included as a study characteristic in a mixed-effects multivariate meta-analysis.

```
library("metaSEM")

load("airlines2.RData")

## Display the first few cases of the data
head(meta.df)
```

```
##   year        y1         y2          v11           v21          v22
## 1 1987 0.8399835 -0.1628065 3.422971e-07 -9.074985e-07 0.0007853871
## 2 1988 0.8238379 -0.8209367 9.000659e-08 -2.175559e-07 0.0001683489
## 3 1989 0.8538955 -0.8318793 8.189956e-08 -1.677359e-07 0.0001703018
## 4 1990 0.9040965 -0.5749465 7.810274e-08 -1.363034e-07 0.0001441517
## 5 1991 0.8401647 -0.7482133 9.496084e-08 -1.718342e-07 0.0001467529
## 6 1992 0.7990992 -0.4952119 1.003837e-07 -1.458418e-07 0.0001522432
```

```
## Meta-analyze results by using a random-effects meta-analysis
## y1: regression coefficient of DepDelay
## y2: regression coefficient of Distance/1000
REM.reg <- meta(y=cbind(y1,y2), v=cbind(v11,v21,v22), data=meta.df,
                model.name="Regression analysis REM")

summary(REM.reg)
```

```
##
## Call:
## meta(y = cbind(y1, y2), v = cbind(v11, v21, v22), data = meta.df,
##     model.name = "Regression analysis REM")
##
## 95% confidence intervals: z statistic approximation
## Coefficients:
##               Estimate   Std.Error      lbound      ubound z value
## Intercept1  0.90107961  0.01761020  0.86656426  0.93559496 51.1681
## Intercept2 -0.86237240  0.10475887 -1.06769601 -0.65704880 -8.2320
## Tau2_1_1    0.00682255  0.00205709  0.00279072  0.01085438  3.3166
## Tau2_2_1   -0.01779759  0.00944805 -0.03631543  0.00072024 -1.8837
## Tau2_2_2    0.24127103  0.07280819  0.09856961  0.38397246  3.3138
##             Pr(>|z|)
## Intercept1 < 2.2e-16 ***
## Intercept2  2.22e-16 ***
## Tau2_1_1   0.0009112 ***
## Tau2_2_1   0.0596012 .
## Tau2_2_2   0.0009204 ***
## ---
## Signif. codes:  0 '***' 0.001 '**' 0.01 '*' 0.05 '.' 0.1 ' ' 1
##
## Q statistic on the homogeneity of effect sizes: 2864828
```

```
## Degrees of freedom of the Q statistic: 42
## P value of the Q statistic: 0
##
## Heterogeneity indices (based on the estimated Tau2):
##                              Estimate
## Intercept1: I2 (Q statistic)   1.0000
## Intercept2: I2 (Q statistic)   0.9995
##
## Number of studies (or clusters): 22
## Number of observed statistics: 44
## Number of estimated parameters: 5
## Degrees of freedom: 39
## -2 log likelihood: -20.8179
## OpenMx status1: 0 ("0" or "1": The optimization is considered fine.
## Other values may indicate problems.)
```

```r
## Variance components of the random effects
VarComp.reg <- vec2symMat(coef(REM.reg, select="random"))

## Correlation between the random effects
cov2cor(VarComp.reg)
```

```
##            [,1]       [,2]
## [1,]  1.0000000 -0.4386675
## [2,] -0.4386675  1.0000000
```

```r
## Plot the effect sizes
plot(REM.reg, axis.labels=c("Regression coefficient DepDelay",
                            "Regression coefficient Distance"),
     ylim=c(-2.5,0.5), xlim=c(0.65,1.1), study.min.cex = 0.6)
```

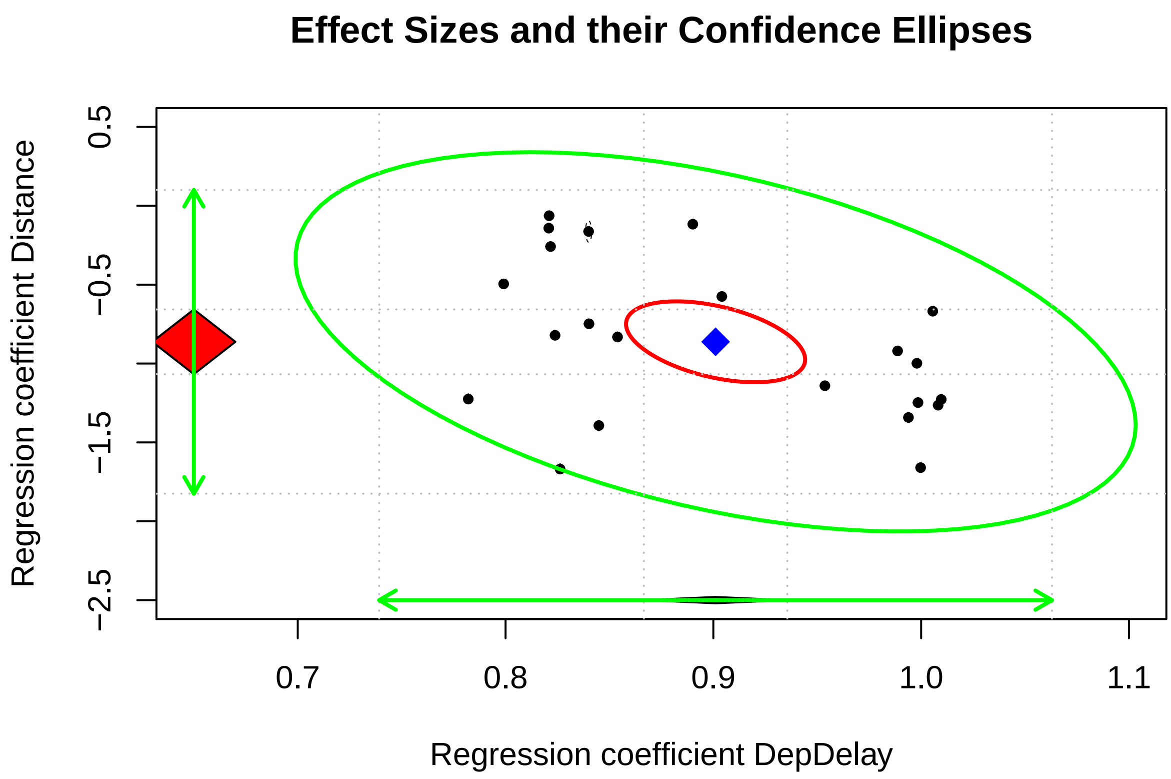


```
## Mixed effects meta-analysis with year as moderator
## year was centered before the analysis.
REM.reg_mod <- meta(y=cbind(y1,y2), v=cbind(v11,v21,v22),
                    x = scale(year, scale=FALSE), data=meta.df,
                    model.name="Regression analysis REM with year as moderator")

summary(REM.reg_mod)
```

```
##
## Call:
## meta(y = cbind(y1, y2), v = cbind(v11, v21, v22), x = scale(year,
##     scale = FALSE), data = meta.df, model.name = "Regression analysis REM with year as moderator")
##
## 95% confidence intervals: z statistic approximation
## Coefficients:
##               Estimate   Std.Error      lbound      ubound z value
## Intercept1  0.90107915  0.01051389  0.88047231  0.92168599 85.7037
## Intercept2 -0.86232952  0.08655247 -1.03196924 -0.69268979 -9.9631
## Slope1_1    0.01044444  0.00165723  0.00719634  0.01369255  6.3024
## Slope2_1   -0.04364979  0.01364415 -0.07039183 -0.01690776 -3.1992
## Tau2_1_1    0.00243185  0.00073325  0.00099470  0.00386900  3.3165
## Tau2_2_1    0.00055044  0.00427016 -0.00781891  0.00891979  0.1289
## Tau2_2_2    0.16464296  0.04969089  0.06725061  0.26203532  3.3133
##             Pr(>|z|)
## Intercept1 < 2.2e-16 ***
## Intercept2 < 2.2e-16 ***
```

```
## Slope1_1   2.931e-10 ***
## Slope2_1   0.0013783 **
## Tau2_1_1   0.0009115 ***
## Tau2_2_1   0.8974334
## Tau2_2_2   0.0009219 ***
## ---
## Signif. codes:  0 '***' 0.001 '**' 0.01 '*' 0.05 '.' 0.1 ' ' 1
##
## Q statistic on the homogeneity of effect sizes: 2864828
## Degrees of freedom of the Q statistic: 42
## P value of the Q statistic: 0
##
## Explained variances (R2):
##                               y1     y2
## Tau2 (no predictor)    0.0068225 0.2413
## Tau2 (with predictors) 0.0024318 0.1646
## R2                     0.6435572 0.3176
##
## Number of studies (or clusters): 22
## Number of observed statistics: 44
## Number of estimated parameters: 7
## Degrees of freedom: 37
## -2 log likelihood: -47.23554
## OpenMx status1: 0 ("0" or "1": The optimization is considered fine.
## Other values may indicate problems.)
```

Mixed-effects model

- A mixed-effects model is fitted to account for the nested structure of the data. The seasonal variation is approximately accounted for by considering the data nested within *Month*, *Day of Month*, *Day Of Week*, while geographical differences is approximately accounted for by considering the data nested within *origin* and *destination* airports.

Read and process data from the database

- Since it takes some time to process large data, the database file is read only if the R data, which has been saved before, is not available.

```r
## Library to read SQLite data and process the data
library("dplyr")
library("lme4")

## Read the SQLite data only if the R image is not available
if (!file.exists("airlines3.Rdata")) {

  ## Read the SQLite data
  my.db <- src_sqlite("1987_2008.sqlite")

  ## Read the table "ontime" into "my.df"
  my.df <- tbl(my.db, "ontime")
```

```
  ## Function to fit regression analysis
  ## y1 to y3: Intercept, DepDelay and Distance/1000.
  ## v11 to v33: Sampling covariance matrix of the parameter estimates
  fun.lmer <- function(dt) {  fit <- try(lmer(ArrDelay~DepDelay+I(Distance/1000)+
                                           (1|Month)+(1|DayofMonth)+(1|DayOfWeek)+
                                           (1|Origin)+(1|Dest),
                                           REML=FALSE, na.action="na.omit",
                                           data=dt), silent=TRUE)
                              if (is.element("try-error", class(fit))) {
                                c(y1=NaN, y2=NaN, v11=NaN, v21=NaN, v22=NaN)
                                } else {
                                ## regression coefficients excluding the intercept
                                y <- unname(fixef(fit)[-1])
                                ## sampling variance covariance matrix excluding the intercept
                                v <- vcov(fit)[-1, -1]
                                c(y1=y[1], y2=y[2], v11=v[1,1],v21=v[2,1],v22=v[2,2])}}

  ## A list of effect sizes and their sampling covariance matrices
  my.list <- list()
  years <- 1987:2008

  for (i in 1:length(years)) {
    my.tbl0 <- filter(my.df, Year==years[i])
    my.tbl1 <- select(my.tbl0, Year, Month, DayofMonth, DayOfWeek, ArrDelay,
                      DepDelay, Origin, Dest, Distance)
    my.data <- collect(my.tbl1)
    my.list[[i]] <- fun.lmer(my.data)
    cat("Completed year: ", years[i], "\n")
  }

  ## Convert my.list into a data frame
  meta.df <- data.frame(Year=years, t(sapply(my.list, function(x) x)))

  ## Save it to avoid rerunning it again
  save(meta.df, file="airlines3.Rdata")
}
```

Conducting a multivariate random-effects meta-analysis

- The regression coefficients on `DepDelay` (y1) and on `Distance` (y2) are considered as multiple effect sizes.

```
library("metaSEM")

## library("OpenMx", lib.loc="~/local/Rlib_github")
## library("metaSEM", lib.loc="~/local/Rlib_github")

load("airlines3.Rdata")

## Display the first few cases of the data
head(meta.df)
```

```
##   Year          y1          y2          v11            v21            v22
```

```
## 1 1987 0.8286496 -0.5824089 3.480258e-07 -5.094999e-07 0.0012361358
## 2 1988 0.8173755 -0.7367829 8.940853e-08 -1.668659e-07 0.0002617783
## 3 1989 0.8457932 -0.9281016 8.217696e-08 -1.358613e-07 0.0002676663
## 4 1990 0.8966704 -0.9644476 7.888457e-08 -1.017208e-07 0.0002214867
## 5 1991 0.8365080 -1.0918253 9.493641e-08 -8.804085e-08 0.0002286743
## 6 1992 0.7905919 -0.9415450 9.989381e-08 -9.750608e-08 0.0002336622
```

```
## Meta-analyze results by using a random-effects meta-analysis
## y1: regression coefficient of DepDelay
## y2: regression coefficient of Distance/1000
meta.rem <- meta(y=cbind(y1,y2), v=cbind(v11,v21,v22), data=meta.df,
                 model.name="Random effects model")
summary(meta.rem)
```

```
##
## Call:
## meta(y = cbind(y1, y2), v = cbind(v11, v21, v22), data = meta.df,
##     model.name = "Random effects model")
##
## 95% confidence intervals: z statistic approximation
## Coefficients:
##              Estimate  Std.Error     lbound     ubound  z value  Pr(>|z|)
## Intercept1  0.8961146  0.0179700  0.8608940  0.9313353  49.8671 < 2.2e-16
## Intercept2 -1.2009773  0.0975517 -1.3921751 -1.0097795 -12.3112 < 2.2e-16
## Tau2_1_1    0.0071042  0.0021420  0.0029059  0.0113025   3.3166 0.0009112
## Tau2_2_1   -0.0153667  0.0088504 -0.0327132  0.0019798  -1.7363 0.0825155
## Tau2_2_2    0.2091064  0.0631470  0.0853406  0.3328722   3.3114 0.0009282
##
## Intercept1 ***
## Intercept2 ***
## Tau2_1_1   ***
## Tau2_2_1   .
## Tau2_2_2   ***
## ---
## Signif. codes:  0 '***' 0.001 '**' 0.01 '*' 0.05 '.' 0.1 ' ' 1
##
## Q statistic on the homogeneity of effect sizes: 2957516
## Degrees of freedom of the Q statistic: 42
## P value of the Q statistic: 0
##
## Heterogeneity indices (based on the estimated Tau2):
##                               Estimate
## Intercept1: I2 (Q statistic)    1.0000
## Intercept2: I2 (Q statistic)    0.9991
##
## Number of studies (or clusters): 22
## Number of observed statistics: 44
## Number of estimated parameters: 5
## Degrees of freedom: 39
## -2 log likelihood: -22.16464
## OpenMx status1: 0 ("0" or "1": The optimization is considered fine.
## Other values may indicate problems.)
```

```
## Variance component of the random effects
VarComp.lmer <- vec2symMat(coef(meta.rem, select="random"))

## Correlation between the random effects
cov2cor(VarComp.lmer)
```

```
##            [,1]       [,2]
## [1,]  1.0000000 -0.3986936
## [2,] -0.3986936  1.0000000
```

```
plot(meta.rem, axis.labels=c("Regression coefficient on DepDelay",
                             "Regression coefficient on Distance"),
     ylim=c(-2.5,0), xlim=c(0.65,1.1), study.min.cex = 0.6)
```

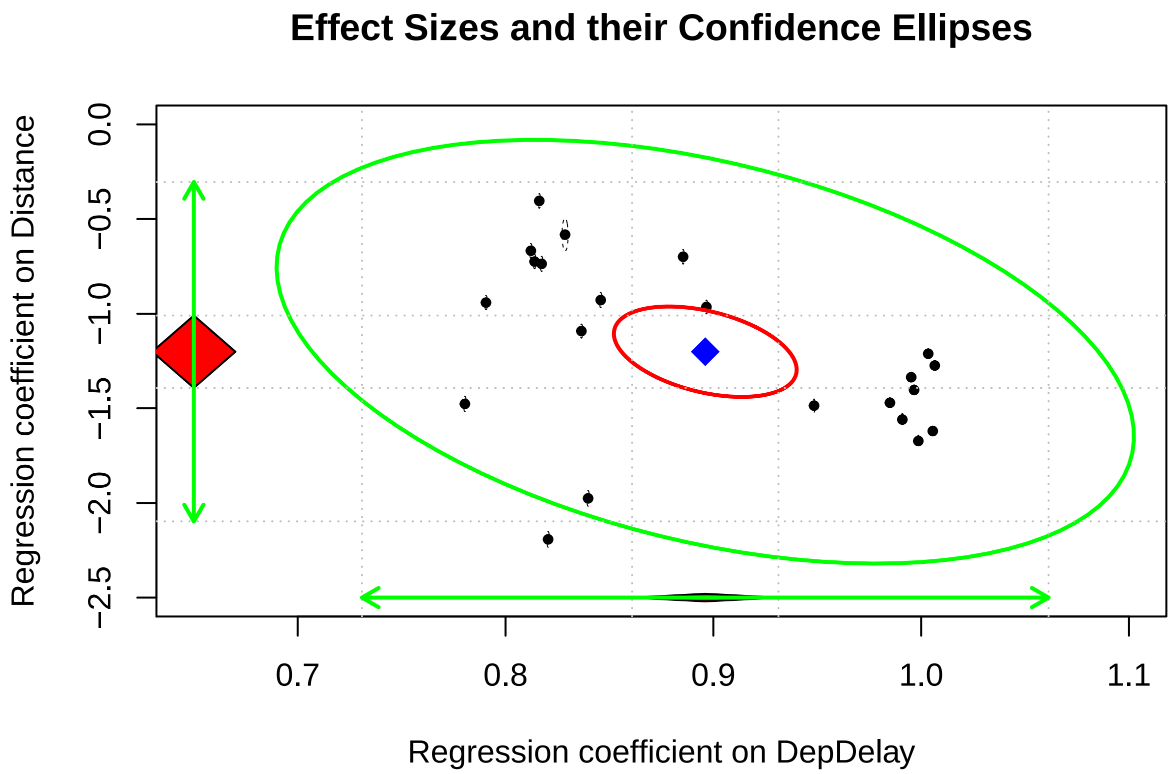

Conducting a multivariate mixed-effects meta-analysis

- A multivariate mixed-effects meta-analysis is conducted by using `Year` as the moderator.

```
## Meta-analyze results with a mixed-effects meta-analysis with year as a predictor
## It may be necessary to use better starting values
## since the variances of the variance components are very different.
meta.mem <- meta(y=cbind(y1,y2), v=cbind(v11,v21,v22), data=meta.df,
                 RE.startvalues=c(0.1,1), x=scale(Year, scale=FALSE),
```

```r
                 model.name="Mixed effects model with year as a predictor")
summary(meta.mem)
```

```
##
## Call:
## meta(y = cbind(y1, y2), v = cbind(v11, v21, v22), x = scale(Year,
##     scale = FALSE), data = meta.df, RE.startvalues = c(0.1, 1),
##     model.name = "Mixed effects model with year as a predictor")
##
## 95% confidence intervals: z statistic approximation
## Coefficients:
##                Estimate   Std.Error      lbound      ubound  z value
## Intercept1  0.89611416  0.01052417  0.87548716  0.91674117  85.1482
## Intercept2 -1.20087470  0.07733268 -1.35244396 -1.04930544 -15.5287
## Slope1_1    0.01076877  0.00165885  0.00751748  0.01402005   6.4917
## Slope2_1   -0.04399448  0.01219204 -0.06789045 -0.02009852  -3.6085
## Tau2_1_1    0.00243661  0.00073469  0.00099665  0.00387657   3.3165
## Tau2_2_1    0.00369812  0.00389829 -0.00394239  0.01133863   0.9487
## Tau2_2_2    0.13131463  0.03967205  0.05355884  0.20907042   3.3100
##              Pr(>|z|)
## Intercept1 < 2.2e-16 ***
## Intercept2 < 2.2e-16 ***
## Slope1_1   8.487e-11 ***
## Slope2_1   0.0003080 ***
## Tau2_1_1   0.0009115 ***
## Tau2_2_1   0.3427982
## Tau2_2_2   0.0009329 ***
## ---
## Signif. codes:  0 '***' 0.001 '**' 0.01 '*' 0.05 '.' 0.1 ' ' 1
##
## Q statistic on the homogeneity of effect sizes: 2957516
## Degrees of freedom of the Q statistic: 42
## P value of the Q statistic: 0
##
## Explained variances (R2):
##                                y1     y2
## Tau2 (no predictor)    0.0071042 0.2091
## Tau2 (with predictors) 0.0024366 0.1313
## R2                     0.6570199 0.3720
##
## Number of studies (or clusters): 22
## Number of observed statistics: 44
## Number of estimated parameters: 7
## Degrees of freedom: 37
## -2 log likelihood: -53.09097
## OpenMx status1: 0 ("0" or "1": The optimization is considered fine.
## Other values may indicate problems.)
```

Settings of the R system

```r
sessionInfo()
```

```
## R version 3.2.5 (2016-04-14)
## Platform: x86_64-w64-mingw32/x64 (64-bit)
## Running under: Windows 7 x64 (build 7601) Service Pack 1
##
## locale:
## [1] LC_COLLATE=English_United States.1252
## [2] LC_CTYPE=English_United States.1252
## [3] LC_MONETARY=English_United States.1252
## [4] LC_NUMERIC=C
## [5] LC_TIME=English_United States.1252
##
## attached base packages:
## [1] parallel  stats     graphics  grDevices utils     datasets  methods
## [8] base
##
## other attached packages:
##  [1] metaSEM_0.9.8     OpenMx_2.5.2      Rcpp_0.12.4
##  [4] Matrix_1.2-6      MASS_7.3-45       digest_0.6.9
##  [7] lavaan_0.5-20     semPlot_1.0.1     R.utils_2.3.0
## [10] R.oo_1.20.0       R.methodsS3_1.7.1
##
## loaded via a namespace (and not attached):
##  [1] splines_3.2.5       ellipse_0.3-8       gtools_3.5.0
##  [4] Formula_1.2-1       stats4_3.2.5        latticeExtra_0.6-28
##  [7] d3Network_0.5.2.1   yaml_2.1.13         lisrelToR_0.1.4
## [10] pbivnorm_0.6.0      lattice_0.20-33     quantreg_5.21
## [13] quadprog_1.5-5      chron_2.3-47        RColorBrewer_1.1-2
## [16] ggm_2.3             minqa_1.2.4         colorspace_1.2-6
## [19] htmltools_0.3.5     plyr_1.8.3          psych_1.6.4
## [22] XML_3.98-1.4        SparseM_1.7         corpcor_1.6.8
## [25] scales_0.4.0        whisker_0.3-2       glasso_1.8
## [28] sna_2.3-2           jpeg_0.1-8          fdrtool_1.2.15
## [31] lme4_1.1-12         MatrixModels_0.4-1  huge_1.2.7
## [34] arm_1.8-6           rockchalk_1.8.101   mgcv_1.8-12
## [37] car_2.1-2           ggplot2_2.1.0       nnet_7.3-12
## [40] pbkrtest_0.4-6      mnormt_1.5-4        survival_2.39-3
## [43] magrittr_1.5        evaluate_0.9        nlme_3.1-127
## [46] foreign_0.8-66      tools_3.2.5         data.table_1.9.6
## [49] formatR_1.4         stringr_1.0.0       munsell_0.4.3
## [52] cluster_2.0.4       sem_3.1-7           grid_3.2.5
## [55] nloptr_1.0.4        rjson_0.2.15        igraph_1.0.1
## [58] rmarkdown_0.9.6     boot_1.3-18         mi_1.0
## [61] gtable_0.2.0        abind_1.4-3         reshape2_1.4.1
## [64] qgraph_1.3.3        gridExtra_2.2.1     knitr_1.13
## [67] Hmisc_3.17-4        stringi_1.0-1       matrixcalc_1.0-3
## [70] rpart_4.1-10        acepack_1.3-3.3     png_0.1-7
## [73] coda_0.18-1
```
